# Optimizing Advance Care Planning in Dementia: Recommendations From a 33-Country Delphi Study

**DOI:** 10.1016/j.jpainsymman.2025.02.471

**Published:** 2025-03-01

**Authors:** Jenny T. van der Steen, Lieve Van den Block, Miharu Nakanishi, Karen Harrison Dening, Deborah Parker, Philip Larkin, Paola Di Giulio, Jürgen in der Schmitten, Rebecca L. Sudore, Ninoslav Mimica, Iva Holmerova, Sandra Martins Pereira, Ida J. Korfage

**Affiliations:** Department of Public Health and Primary Care, Leiden University Medical Center, Leiden, The Netherlands; Department of Primary and Community Care, Radboud University Medical Center, Nijmegen, The Netherlands and Cicely Saunders Institute, King’s College London, UK; VUB-UGent End-of-Life Care Research Group, Jette, Brussels, Belgium; Department of Public Health and Primary Care, Leiden University Medical Center, Leiden, The Netherlands; Department of Psychiatric Nursing, Tohoku University Graduate School of Medicine, Sendai-shi Miyagy, Japan; Research & Publications, Dementia UK, London, UK; Faculty of Health & Life Sciences, De Montfort University, Leicester, UK; IMPACCT/School of Nursing and Midwifery, Faculty of Health, University of Technology, NSW, Sydney, Australia; Palliative and Supportive Care Service and Institute of Higher Education and Research in Healthcare, UNIL | Université de Lausanne, CHUV | Centre hospitalier universitaire Vaudois, Faculté de biologie et de médecine – FBM Institut universitaire de formation et de recherche en soins – Hôpital Nestlé, Lausanne, Switzerland; Department of Public Health Sciences and Pediatrics, Turin University, Turin, Italy; Institute for General Practice/Family Medicine, Medical Faculty, University of Duisburg-Essen, Essen, Germany; Division of Geriatrics, Department of Medicine, University of California, San Francisco, CA, USA; San Francisco Veterans Affairs Medical Center, San Francisco, CA, USA; University Psychiatric Hospital Vrapče, School of Medicine, University of Zagreb, Zagreb, Croatia; Faculty of Humanities, Centre of Expertise in Longevity and Long-Term Care and Centre of Gerontology, Charles University, Prague, Czech Republic; Universidade Católica Portuguesa, CEGE: Research Center in Management and Economics – Ethics and Sustainability Research Area, Catolica Porto Business School, Porto, Portugal; Department of Public Health, Erasmus MC, University Medical Center Rotterdam, Rotterdam, The Netherlands

**Keywords:** Advance care planning, decision making, dementia, end of life, guidelines, palliative care

## Abstract

**Context.:**

Advance care planning (ACP) is relevant yet challenging with cognitive decline.

**Objective.:**

To provide evidence and consensus-based clinical recommendations for how to conduct ACP in dementia.

**Methods.:**

International Delphi study conducted by the European Association for Palliative Care ‘ACP in dementia’ taskforce with four online surveys (September 2021-June 2022). A panel of 107 experts from 33 countries and seven individuals with dementia contributed. The recommendations specific for dementia were initially based on two earlier Delphi studies and literature searches addressing guidance including the right timing and how to personalize ACP. We used conservative preregistered criteria for consensus.

**Results.:**

Thirty constitutive elements of ACP were identified (e.g., ‘assess understanding of ACP’). Only five were deemed ‘optional.’ The panel estimated a median of four conversations could address elements to be addressed at least once. Recommendations included to assume capacity as a principle, conscious of the need to explore its fluctuation, to encourage engaging and playing active roles, and to establish connection and inform and prepare family. There was a consensus to offer ACP around dementia diagnosis, to raise end-of-life issues later, and to personalize ACP with flexibility, providing of information and exploring understanding. The advice of the persons with dementia pointed to a wish for a well-coordinated holistic approach.

**Conclusion.:**

Consensus was reached, including in areas of ambiguity, to guide ACP in dementia. ACP should be embedded in a nonprescriptive, individualized approach that involves both the person with dementia and their families. Future studies may evaluate trade-offs between optimal ACP and feasible implementation.

## Introduction

Advance care planning (ACP) has been advocated as a process to enable individuals to express wishes for future care needs ahead of capacity loss and can facilitate proxy decision making when the need to decide about care and treatment arises.^1–3^ There are different approaches, methods, conceptualizations and goals of ACP itself.^4,5^ Results in terms of its outcomes are conflicting, which has triggered persistent debates about the value and effectiveness of ACP and its outcomes and to what ACP and related communication approaches actually aspire (e.g.),^6,7^ usually referring to after-capacity loss.

**Fig. 1.**
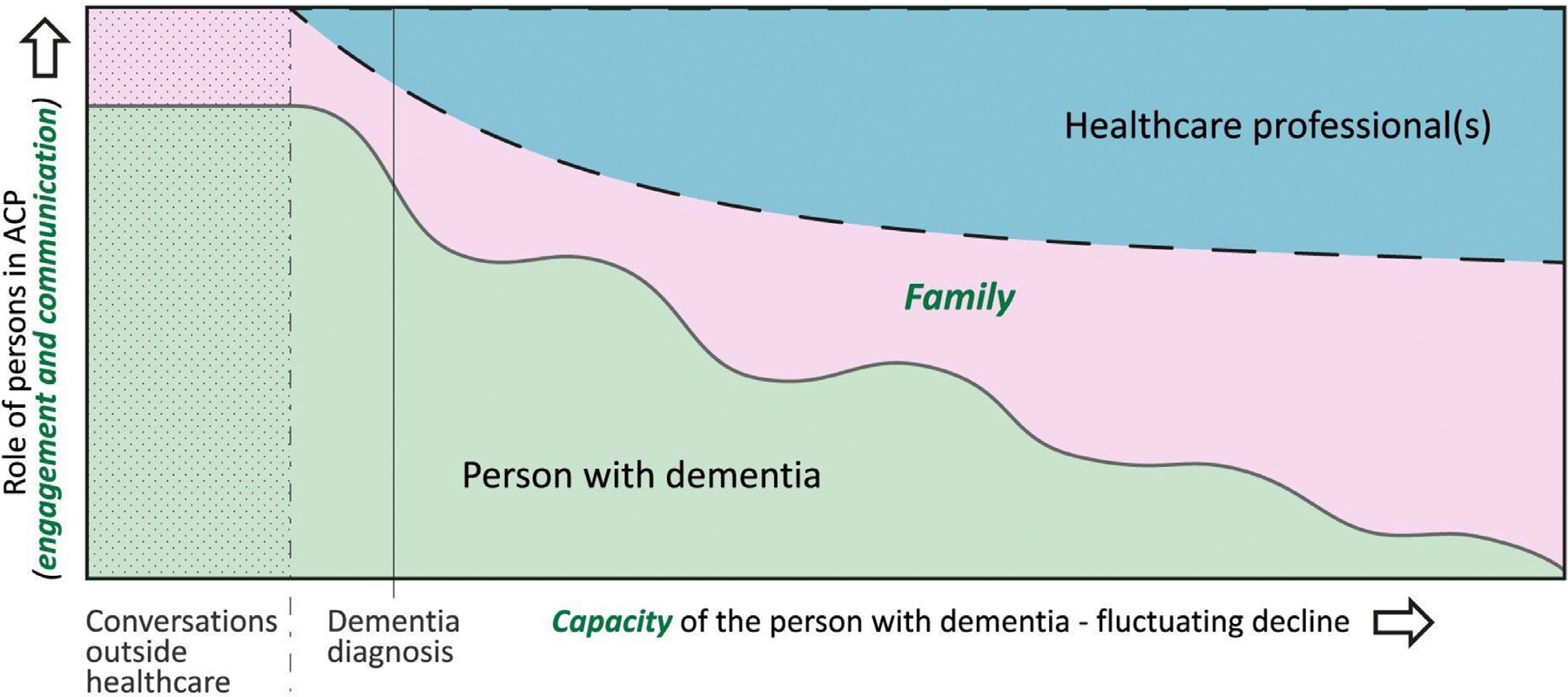
Relating the three issues specific to Advance care planning (ACP) in dementia and change over time. The Fig. shows how three dementia-specific issues (green text) that are of particular importance in the case of dementia in ACP may relate and change with dementia progression during the ACP process. It indicates an ideal model of the engagement in ACP of the person with dementia as long as possible given an unavoidable decline in capacity, along with engagement of the family who is available and involved in the ideal situation, and health care professional(s) with whom the person has trusting relationships. Shaded green indicates conversations outside health care. The green area shows the typical declining contribution and fluctuating active role played (Y axis) of the person with dementia due to decline in capacity (X axis), and the other areas show how this may influence active roles played in ACP by family and health care professional(s). Disclaimer: there are many other factors that influence roles in ACP, while the model cannot show its complexities or detail. Reproduced from: van der Steen JT et al.; EAPC, Alzheimer’s & dementia: the journal of the Alzheimer’s Association 2024;20(2):1309–1320. doi: 10.1002/alz.13526.

Frequently cited definitions of ACP^8,9^ have explicitly limited ACP to persons with (full) decision-making capacity. However, ACP is of no less relevance for people with cognitive decline. Indeed, the Lancet dementia commission stated that ACP, “designed to empower people with dementia and improve quality of dying, might theoretically be something everyone should do before developing dementia” yet it is inherently complicated, for example due to inability to predict future wishes.^10,11^ In the case of dementia, capacity may fluctuate but inevitably decreases in the long term.^2,12^ This necessitates increasing involvement of family or others, and adaptation of communication tailored to enable engagement in the process, as capacity allows.^13^ In a Delphi study, our taskforce of the European Association for Palliative Care (EAPC) and an expert panel recently defined ACP in dementia as a relational and person-centred way to elicit preferences, values and goals, and to be continued when decision-making capacity diminishes.^14^ Further, a consensus was reached on capacity, family, and engagement/communication as being three related issues of particular importance to ACP in dementia (Fig. 1).

The consensual framework of a definition and a description of these three issues provides a basis of what ACP in dementia aspires to but does not offer guidance for practitioners on its contents or process. Practice guidance for professionals is needed on what elements of ACP should constitute a first conversation and, realistically, follow-up conversations. Few trials have been conducted, but the literature is replete with dilemmas around ACP and reported barriers to ACP, including a number of reviews of barriers, also in dementia (e.g., reviews^2,15^) and often point to the need for improved guidance on ACP in dementia (e.g., reviews^16–18^). Indeed, the evidence on how to conduct ACP in dementia is fragmented, and the literature usually draws on sources from a single high-income country (for example, in preparatory work in 2021,^19^ we identified 13 randomized controlled trials, all from single western countries and most from the US). What surfaces is that the right timing and tailoring of contents to individual needs because of different levels of readiness are crucial, especially in the case of dementia. This implies that ACP in dementia in particular needs to move away from decisions taken exclusively in advance of capacity loss or situations that might arise. Comprehensive guidance could inspire and support healthcare professionals in conducting ACP with persons with dementia and their family caregivers. Therefore, we involved experts from across the globe, and persons with dementia themselves, to integrate available evidence with consensus-based practice recommendations for professionals on optimal content and process of ACP for persons with dementia and their families.

## Methods

### Design

We designed a Delphi study to achieve international consensus on optimizing ACP in dementia. The objective reported in the study protocol^19^ was “To conceptualise ACP in dementia in terms of its definition, elements, and any differences with ACP in patients with other diseases who are expected to retain capacity. Further, based on evidence and consensus, to provide recommendations to optimize ACP in practice, for policy initiatives to promote ACP in dementia, and for areas that need research.” Following publications on the differential definitional framework,^14^ and recommendations for policy and research,^20^ in this core article, we report on the key clinical issues of content (elements) and process of optimal ACP in practice.

**Fig. 2.**
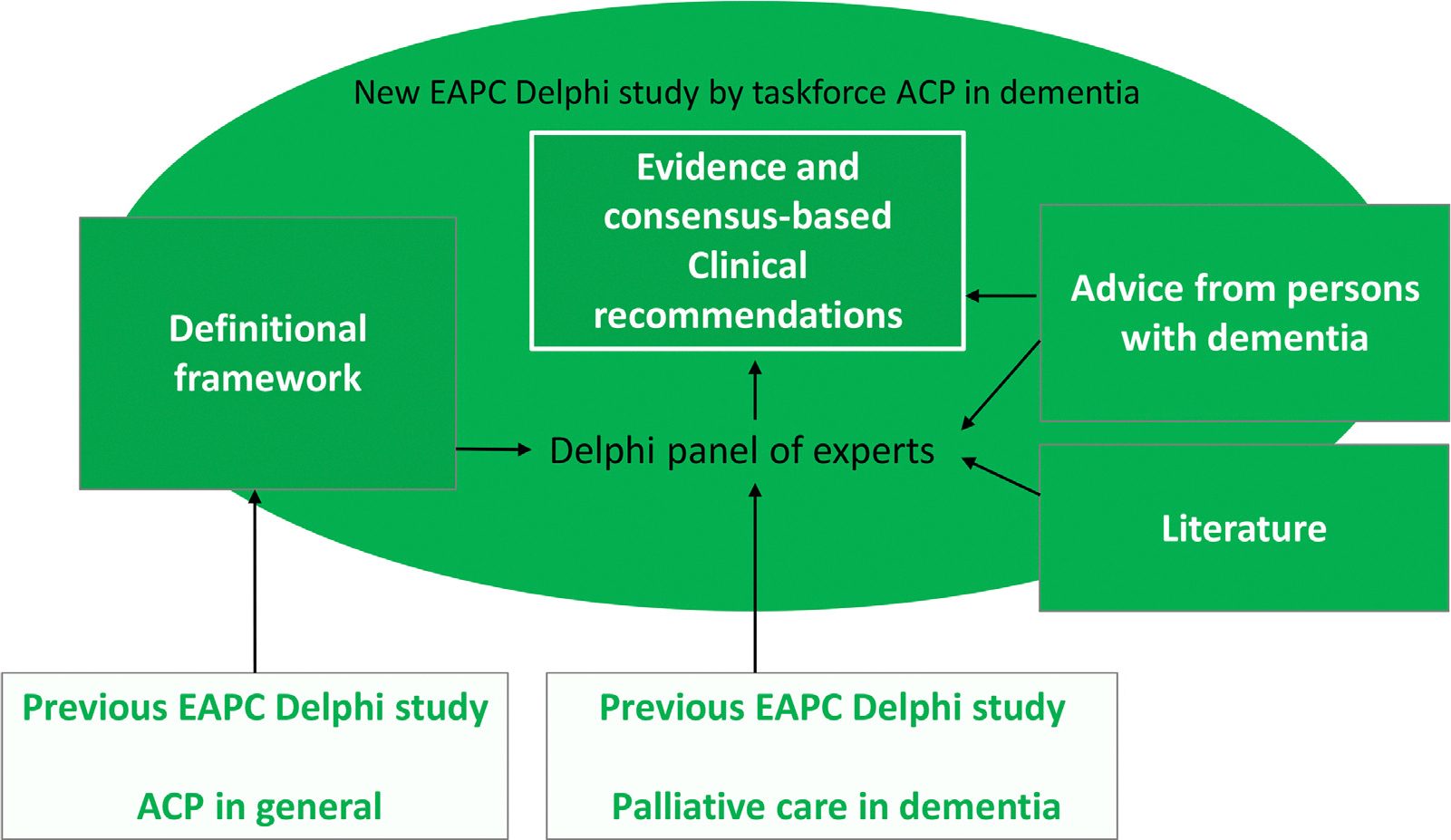
Building up the evidence and consensus based clinical guidance on ACP in dementia: Methods and sources. The evidence and consensus-based clinical recommendations represent the new, core end product presented in this manuscript, adding to other products of the task force answering the other two research questions: a definitional framework^14^ and recommendations for policy & research^20^ A detailed version of this Fig. is included in the Methods Supplement (number 3). ACP = advance care planning; EAPC = European Association for Palliative Care.

Supplement A reports detail on the methods, with phases and rounds anticipated vs. conducted in Supplement A1 and A2. The recommendations for clinical practice were based on multiple sources, iteratively integrating evidence from the literature and expert input (Fig. 2; Suppl A3 and A4). In the final phase of the Delphi study, the EAPC Board of Directors reviewed and approved the article.

### Experts

**Fig. 3.**
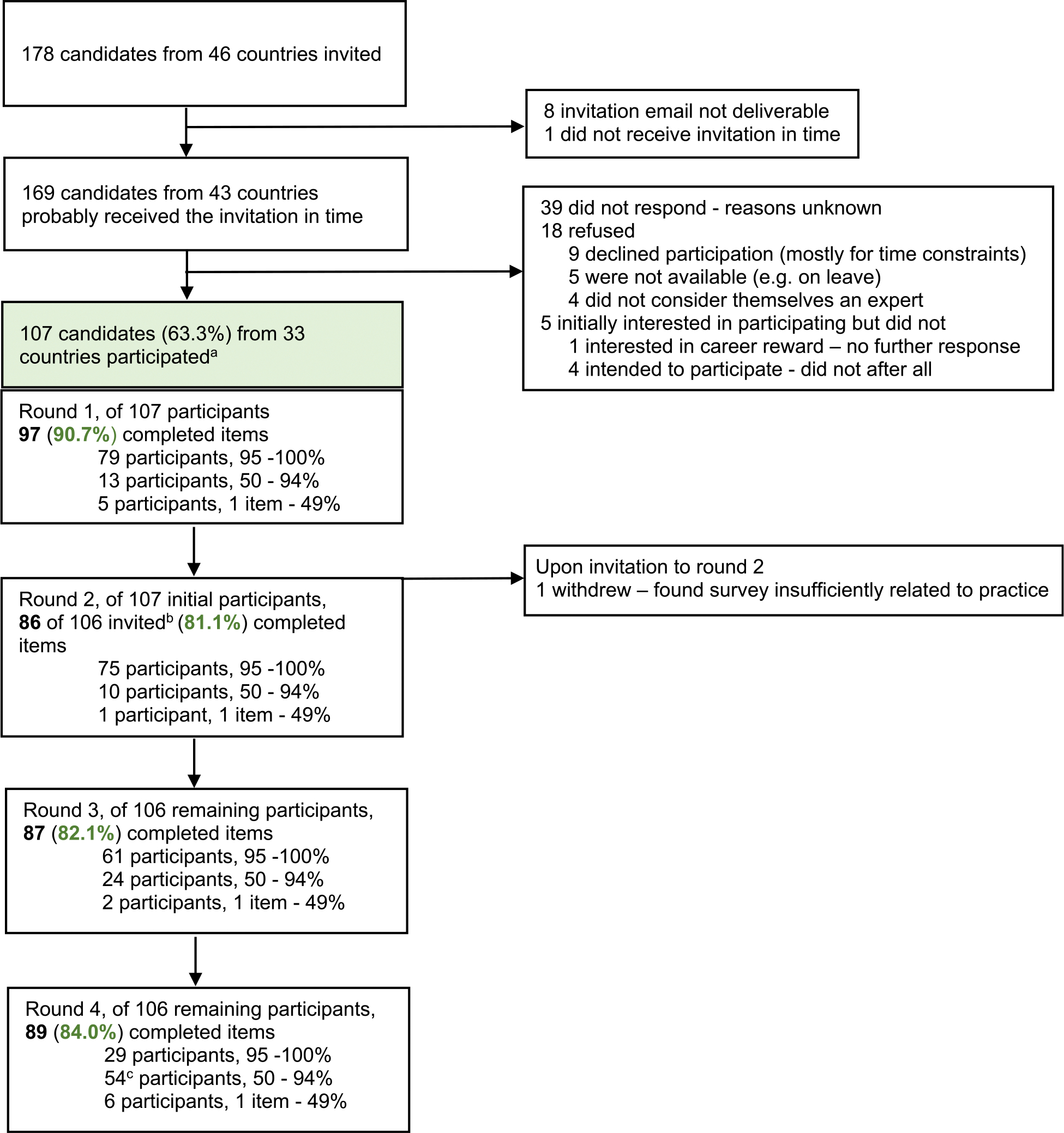
Flow chart participation delphi expert panel and response per survey round. ^a^Participants were defined as those who provided informed consent and completed survey items upon the first or the second invitation (no third invitation was sent to non-respondents). Overall response rate: 107/169 (63.3%) participated. Of the 107 (initial) participants, 11 (10.3%) completed a single round, 8 (7.48%) 2 rounds, 22 (20.6%) 3 rounds, and 66 (61.7%) completed all rounds. ^b^We forgot to send an invite to one of the participants. ^c^Of 54 participants who completed 50–94%, 39 completed 92% which was the maximum percentage when missing a hidden item beneath a long list of possible outcomes for evaluation. Reproduced from: van der Steen JT et al.; EAPC, Alzheimer’s & dementia: the journal of the Alzheimer’s Association 2024;20(2):1309–1320. doi: 10.1002/alz.13526.

The EAPC taskforce ACP in dementia with 14 members from a broad range of countries reviewed previous work and involved a Delphi panel of experts in ACP and dementia care sampled for diversity in country, profession and expertise (Fig. 3; Supplement A5 and A6). Panellists were identified through networks of the taskforce, through having participated in previous Delphi studies, websites from dementia-related organisations, and PubMed searches to find more English-speaking experts from nonwestern countries. Additionally, input from persons with dementia was sought. The Delphi panel evaluated recommendations in four online surveys between September 2021 and June 2022 with survey-round specific response rates between 81.1% (round 2) and 90.7% (round 1) (Fig. 3).

### Developing the Surveys

First, the full taskforce was involved in adapting evidence and consensus regarding ACP more generally for persons with capacity from the earlier EAPC ACP taskforce (Rietjens et al.^8^; bottom left part of Fig. 2; detailed Fig. in Supplement A3). Revising the general recommendations to apply to dementia increased their length. Therefore, we used the revisions as interim results which the core task force members (JTvdS, LVdB, MN and IJK) analysed, abstracting elements of ACP, described as briefly as possible. In the first survey round, we asked the panel to evaluate the brief elements of ACP in dementia and which ones to repeat each time. Response options were: ‘yes, usually each time’; ‘yes, usually once’; and ‘no’. We retained elements that were endorsed (any yes) by at least 80% of the panellists. Based on the ratings, we grouped items and presented three groups to the panel (always, at some point, optional) for evaluation in subsequent rounds. We also asked for any missed elements that should be added using open text boxes. Second, items in the form of recommendations or statements were also inspired by recommendations on ACP based on an earlier Delphi study that included review of the literature reported in the EAPC white paper palliative dementia care^24^ (bottom right part of Fig. 2). Third (upper left part of Fig. 2), further analyses of the adaptations to the guidance on ACP more generally,^8^ we found three issues that deserved particular attention in dementia: ‘capacity’, ‘family’ (in a broad sense, see definitions in the footnote to Table 1), and ‘engagement and communication’. To develop clinical recommendations for each of three issues, three taskforce subgroups (LvdB, KHD, SMP for capacity; MN, DP, JidS, RLS, IJK for family; JTvdS, PL, PDG for engagement and communication) in consultation with experts as needed, reviewed the literature rapidly with targeted searches, or a systematic scoping review of the full issue in the case of engagement and communication for which evidence was sparse.^13^ In consultation with experts, the subgroups created key recommendations using the same structured form for the three issues.

Fourth, in parallel (right part of Fig. 2), drawing on the expertise of the taskforce and 24 reviews abstracted from three meta-reviews^17,21,22^ identified by our literature searches,^19,23^ the core task force identified challenges in the process of ACP, to develop survey items to clarify guidance on finding the right timing (of initiating and follow-up conversations) and on personalizing ACP (adopting a prudent approach, providing information, and exploring understanding). Fifth, the core task force used advice from people with dementia. We created recommendations based on an analysis of interviews with community-dwelling persons with young-onset dementia (<65 years) in Flanders^18^ (summarized advice) and direct advice (“tips”) solicited during interviews on ACP in the Dutch Care4Youngdem study (2019–2022).^25^ We phrased their responses for conciseness and we were able to reach seven of 10 interviewed persons with dementia about their recommendations in 2022, inviting them to verify or comment on the phone. We asked the Delphi expert panel to evaluate the importance of the recommendations from the two interview studies and to what extent they found the tips “eye-opening”-meaning: revealing or surprisingly teaching something new. Persons with dementia were not part of the Delphi panel so as to not dilute their input, and not confront them with lengthy digital self-complete surveys over an extended period. We did not invite family caregivers as such to join the panel^19^ as we expected many panellist to already have family experiences of caring among a typically middle-aged cohort of expert panellists.

For controversies that emerged from the multiple open-ended comments fields in the survey, we developed new items. We avoided a low-risk approach aiming at immediate consensus without examining the exact controversy, to allow for a better understanding followed by targeted revisions and guidance developed in consensus in the rounds afterwards. Therefore, we explored ambiguities (e.g., on raising end of life) and breadth (e.g., also asking about mild cognitive impairment (MCI)).

We used a 5-point agreement scale with coded response options: “1) strongly disagree”, “2) moderately disagree”, “3) neither agree, nor disagree”, “4) moderately agree”, and “5) strongly agree”. Conservative evaluation criteria for consensus (defined as high or very high (dis)agreement) were: very high agreement, a median of 5 and an inter-quartile range (IQR) of 0 and ≥80% scoring a 4 or 5; high agreement, a median 5 and an IQR ≤1 and ≥80% scoring a 4 or 5; moderate agreement, a median of 4–5 and an IQR ≤2 and ≥60% scoring a 4 or 5; low agreement, a median of 4–5, and an (IQR ≤ 2 or ≥ 60% scoring a 4 or 5); no agreement, a median 4–5 otherwise or a median >2 and <4. For consensus on disagreement, we reversed the median with the same IQR requirements and disagreement percentages.^24^

We present clinical recommendations that achieved a consensus, while the process of achieving it along with the survey instrument is detailed in Supplement B1–B5. We developed the surveys in an electronic data capture system (Castor edc, Amsterdam) which included colour visualizations, a menu and other features that supported user-friendliness of long surveys. We refrained from mandatory items, instead programmed warnings if items (other than boxes for additional comments) were left blank. We pilot-tested the electronic surveys with local colleague researchers on ACP in dementia.

### Analyses

Open-ended comments were analysed by JTS and by another researcher (MN, SMP, IJK, or SG). We refrained from three sets of preplanned subgroup analyses (physicians vs other, ACP in dementia specific expertise, and personal experience).^19^ In the analyses of consensus with the definitional framework, the subgroup differences were mostly negligible and without clear patterns that could inspire new hypotheses.^14^ However, for items on young-onset dementia, we conducted straightforward sensitivity analyses describing agreement and consensus in the selected group of those with the expertise (as appropriate; meaning not an evaluation of the advice from the persons with young-onset dementia).

We conducted nonresponse analyses with four relevant characteristics that could be assessed reliably from public profiles: gender, residence in Europe, medical background, and a PhD degree. We tested the comparison between panellists and nonresponding invitees with Chi-square tests.

### Ethics and Reporting

The Medical Research Ethics Committee Leiden-Den Haag-Delft reviewed the study protocol on 2 September 2021 (reference N21.105). Five days afterwards, the protocol of the Delphi study was registered.^19^ The reporting in the article including the two Supplements adheres to the Guidance on Conducting and Reporting Delphi Studies (CREDES).^26^

## Results

### The Panel

The response rate to the online survey was 63.3% (107/169, Fig. 3). The panellists came from 33 countries including 16 outside Europe-the Americas, Asia, the Middle East, Australasia and Africa (Supplement A6).^14,20^ With about half (51.5%; 17/33) of countries situated in Europe, of the panellists, slightly over half (58.9%; 63/107) came from Europe.

Most were women (74, 69.2%) and mean age of the panellists was 52.0 (SD 12.1) years. Half (49.5%) had a medical background (52 physicians and one nurse practitioner or physician assistant). One-fifth was nurse (21, 19.6%); others were psychologists, ethicists, policy/administration workers, social workers, epidemiologists, spiritual counsellors, or had yet another profession such as sociologist, occupational therapist, lawyer, or economist. Over two-thirds (72/102, 70.6%) had personally experienced a family member or friend having advanced dementia at the end of life.

Mean professional experience was 24.4 (SD 11.8) years. In particular (not in Supplement), professional experience concerned experience in research (78.1% in dementia, 71.2% in ACP research), clinical practice (74.3% in dementia, 59.6% in ACP), and/or policy/administration (25.2% in dementia, 20.4% in ACP). Other expertise, such as teaching and ethics, was reported by 5.8% (dementia) and 4.9% (ACP); some reported a lack of ACP (4.7%) or dementia care expertise (8.5%). Over half (57.7%, of 104 responses) reported having expertise in ACP in dementia specifically; 28.8% reported separate expertise in dementia care and in ACP; 8.7% in dementia but not ACP in dementia; and 4.8% in ACP but other than with dementia. Almost three-quarters (74.0%, 104 responses) had specific expertise in palliative care, and 20.4% (of 103 responses) in young-onset dementia.

There were no appreciable differences between the 107 panellists and 62 nonresponders in gender (69.2% vs. 66.1% women, *P* = 0.68); residing in Europe (58.9% vs. 50.0%, *P* = 0.26); medical background (49.5% vs. 43.5%, *P* = 0.45) or having obtained a PhD degree (62.6% vs. 59.7%, *P* = 0.71).

### Elements of ACP

#### Recommended Elements.

**Fig. 4.**
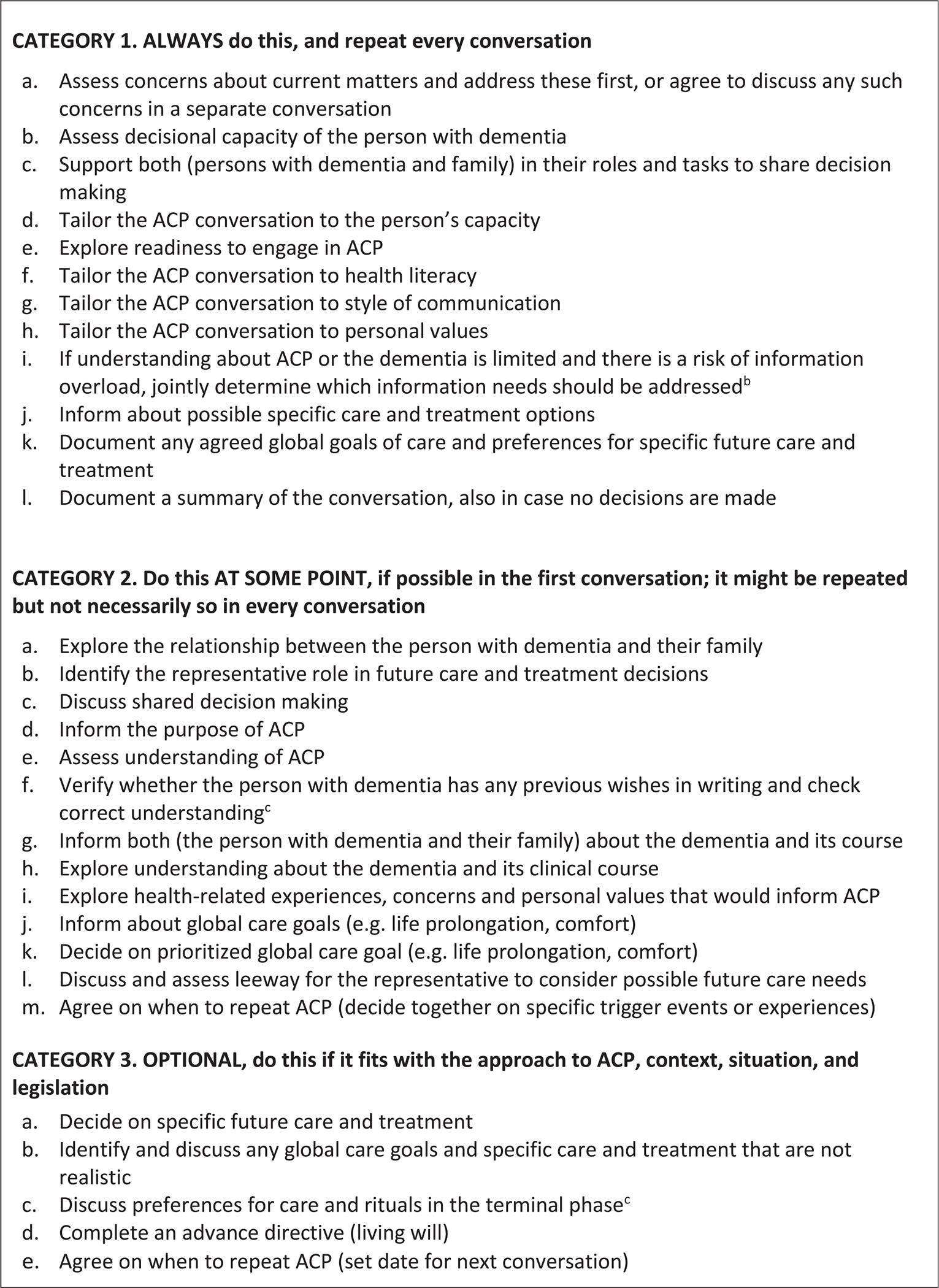
The elements of ACP in dementia by need to repeat in multiple conversations.^a^ ^a^The three categories as a whole reached a consensus assuming that both the person with dementia and family are involved and that multiple conversations are possible. Items refer to elements of the conversation itself. Consensus was reached with high agreement for the three categories (median 5, IQR 1, excluding 1 do not know), and percentages agreement were 88.2% (*n* = 85) for category 1, 91.7 (*n* = 84) for category 2, and 92.7% (*n* = 82) for category 3. ^b^This item was rephrased for clarity after the category as a whole achieved a consensus, to replace the phrase ‘prioritize addressing information needs’. ^c^In round 1, 28 elements were introduced as a list, and the two elements 2f and 3c were added in round 2 based on an emerging public health approach^14^ where conversations can start outside healthcare (2f) and upon suggestions of the panelists (3c). In round 3, adding of these elements reached a consensus with high agreement (2f: median 5, IQR 1, 96.1% agreement, *n* = 76, 3 don’t know; 3c: median 5, IQR 1, 84.6% agreement, *n* = 78, 5 do not know). Supplement B1 provides detail on development over three evaluation rounds. Note that category numbers 1 and 2 were reversed compared to how they were presented to the panel which is in the Supplement. ACP = advance care planning.

Fig. 4 shows a total of 30 brief elements of ACP-mostly behaviours or tasks. Twenty-eight were abstracted from our analyses of adaptations to the generic EAPC ACP recommendations (left of Fig. 2) and two were added in subsequent rounds. In round 1, all 28 were endorsed by at least 80%, yet support was lowest for ‘Date when to repeat’ (19%) and ‘Complete an advance directive’ (18% responded ‘no, not an element’). Supplement B1 details the instrument and feedback shown to the Delphi panel, revisions and evaluations and the achieving of consensus on the elements over three survey rounds.

While individual items were presented to the panel in the first round, we retained all and grouped for the second round, refining the category of ‘usually once’ to become ‘do this at some point, if possible, in the first conversation.’ The addition of ‘Verifying previous wishes and correct understanding’ ‘at some point’ achieved a consensus. There was only moderate agreement (therefore no consensus) about the need of a fourth category for items of ACP conversations about the terminal phase but adding it as an optional item did achieve a consensus.

Feedback from the panellists indicated concern about feasibility. An exemplary comment of a panellist was: “*Regarding the ALWAYS do this statements, I do agree, but I am not sure either are feasible or realistic. They both run the risk of setting up health care professionals for failure, or alienating them, potentially jeopardising the whole process*.”

Therefore, we did not add elements to prepare *before* the actual ACP conversation, while some panellists suggested verifying if there had been any triggers and, ahead of starting ACP conversations, to provide information or recommend the person and family to talk together. We also refrained from adding items such as investing in a trustful relationship, which could be seen as part of an underlying process, and person-centred communication skills more generally. There was no consensus (moderate agreement) about the feasibility of conducting a series of ACP conversations that cover all elements of categories 1 and 2 (still 24 in round 1; Supplement B1). On average, the panellists estimated this would require a median of 4 conversations (IQR 3, range 0–15; *n* = 79).

#### Elements of ACP in Dementia Applied to Other or Specific Populations.

There was no consensus (moderate agreement only) that the elements similarly apply to persons with MCI; panellists citing a different prognosis. Neither would they apply to persons with no dementia, referring to no need to consider capacity or to involve the family with the same necessity. However, some supported broader applicability: “*The items addressed in the categories are excellent areas for consideration for anyone looking to engage in ACP*.”

There was a consensus (high agreement) that the elements of ACP should be the same for persons with young-onset dementia (compared with the elements proposed for a total population of persons with dementia), also among the subgroup of experts with expertise in young-onset dementia specifically (Supplement B1). Some mentioned more social issues needed to be addressed such as children, finances, and less medical issues due to fewer comorbidities yet “*treatments and preferences may differ, but the process should be similar*” or that it is “*often much more difficult*.” When asked in the next round, there was no consensus on whether ACP with young-onset dementia is ‘more difficult’ than ACP in older persons with dementia.

#### Recommendations for the Three Specific Issues.

Table 1 reports the most salient recommendations and the evaluations of the three issues that reached a consensus in being of specific or of particular importance to ACP in dementia. The sets of recommendations on Family (three recommendations; on encouraging, informing and preparing family) and on Engagement and Communication (three on active role, and three on communication issues) immediately achieved consensus, with high agreement.

The recommendations on Capacity needed another round (only moderate agreement initially). There had been two recommendations, referring to capacity testing and avoiding undue influence of others (Supplement B2). The capacity subgroup revised based on the panel’s feedback and additional screening of literature on capacity, ACP and decision-making. This work resulted in very high agreement (median 5, IQR 0, 96.4% agreed) on assuming capacity as a principle, fluctuating and decision-specific capacity and assessment tools not always being necessary.

#### Recommendations on Personalizing ACP.

Table 2 shows recommendations that achieved a consensus on adopting a prudent, flexible approach, providing the right information, and exploring understanding which we summarized as personalizing ACP. Some achieved a consensus with very high agreement with only a few panellists not agreeing. This was true for providing information on the disease and care and treatment (recommendation e) and a long recommendation (a) to adopt a person-centred or individualized approach combined with tailoring elements (as many as 84 of 85 panellists, 98.8% agreed). Recommendations to tailor to capacity (b) and start with discussing current care (c) achieved a consensus with high agreement. Revision was needed (Supplement B3) on what to do in the case of resistance, which, after incorporating feedback, resulted in a longer, more nuanced recommendation (d).

### Timing of ACP

#### Recommendations on Initiating.

Consensus with very high agreement supported people be offered the opportunity to engage in ACP ‘shortly after’ diagnosis (c in Table 3). The same recommendation referring to ‘at diagnosis’ achieved a consensus with high agreement (b) while also people with no dementia but with MCI should be offered the opportunity (a). Consensus with high agreement also supported ‘as soon as the diagnosis is made’ (d), a recommendation that additionally refers to the limited window of opportunity and the implications rather than just ‘offering’ (a-c) which was found ‘nonintrusive’. Importantly, end-of-life care may be discussed as part of ACP a few months after diagnosis (e) while doing so at diagnosis did not achieve a consensus.

**Fig. 5.**
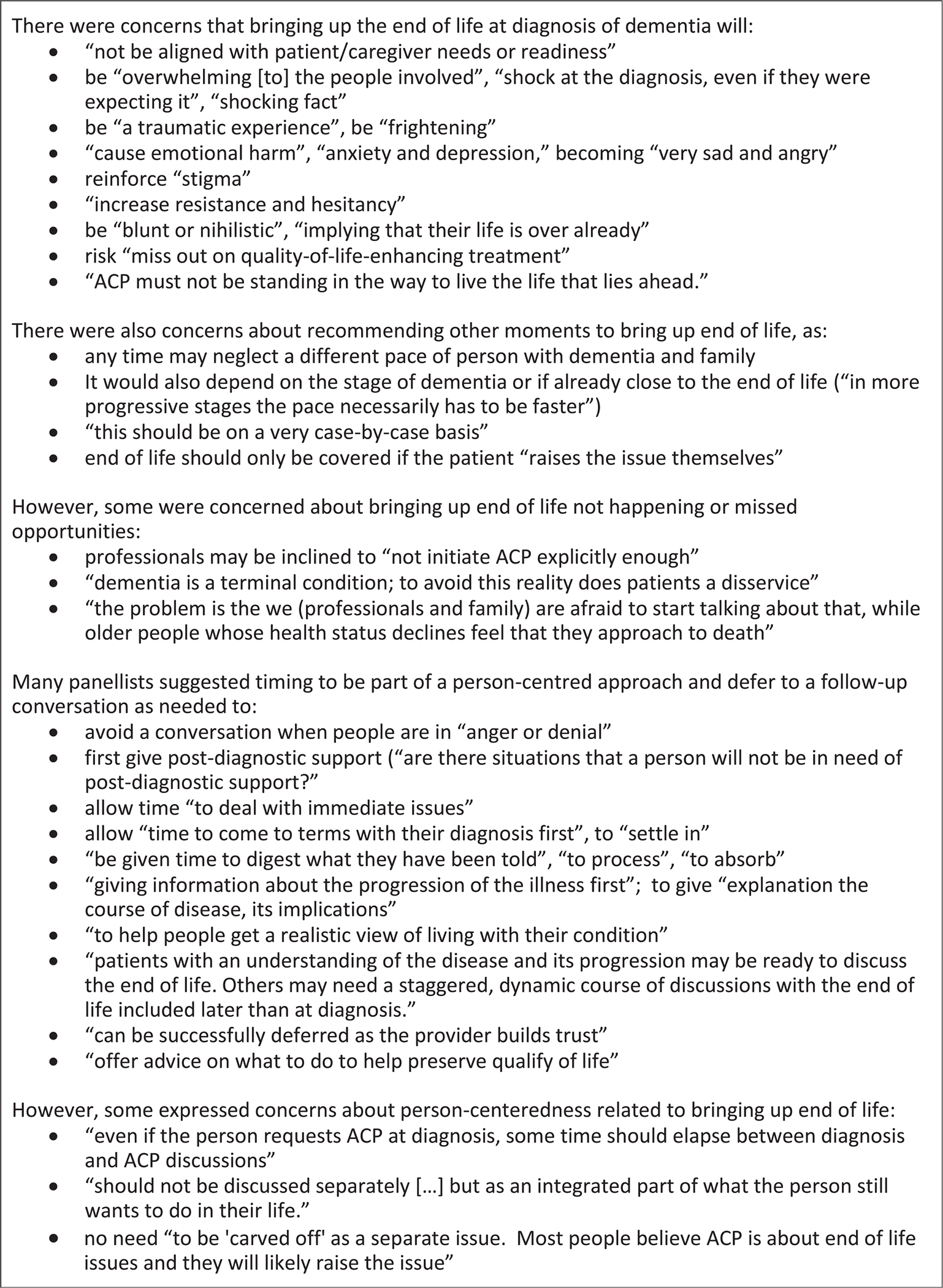
Controversies and concerns around timing of bringing up the end of life expressed by the panellists. ACP = advance care planning.

Fig. 5 reports on the panellists’ multiple comments about bringing up the end of life related to the recommendations on timing that achieved (Table 3) or did not achieve a consensus (Supplement B4). Some panellists expressed concern that bringing it up at diagnosis or on other times will cause emotional harm to the person with dementia and the family, especially when not part of a person-centred approach to ACP. However, there were also concerns that such concern could result in missed opportunities to talk about the end of life.

#### Recommendation and Triggers for Updating.

There was a consensus with high agreement to update plans at least yearly and more frequently as needed (Table 3, (f)). A total of 11 triggers were endorsed by the panel, of which five created based on the panellists’ feedback. Five of the eight that were not endorsed (Supplement B4) referred to family situations. Further, two (not wanting to live, and change in wellbeing) would rather trigger conversations about current care, and end of life was not found to be a clear enough trigger.

### Advice From Persons With Dementia

Table 4 summarizes the advice from persons with young-onset dementia for healthcare professionals conducting ACP with persons with dementia. The full “tips” in context can be read in Supplement B5. Two of the three advices evaluated as the most valuable by 55% of the panellists (Tips 1a and 1c in Table 4) concerned summarized analyses of interviews. The third, valued by 55% of the panellists, was from a person with dementia directly who recommended healthcare professionals to listen carefully to really understand what someone needs (2k).

However, in contrast to the direct personal advice, the summarized advice was not or rarely-for only 0%–2% of the expert panellists-found eye-opening. The most eye-opening direct advice was to raise existential issues as part of ACP, to limit the number of providers, and to look at the person and their capabilities, behind the dementia (2a–2c; 11%–16% of panellists). Some panellists (Supplement B5) reflected on the extent to which tips were generalizable or particular for a culture or individual, for example in how to address existential issues and who will be there during ACP conversations.

## Discussion

As an international taskforce of EAPC, we conducted a Delphi study to optimize ACP in dementia systematically employing accumulating literature and expert consensus commensurate with our mission “to provide people with dementia with equitable opportunity and a voice to identify and articulate what is important to them in their lives and in their relationships with those around them.”^27^ There is a clear need to conceptualise ACP in a way that is inclusive for people with dementia and their families. Future care for persons with dementia involves substantial uncertainties but certain decline in capacities. This capitalizes on the relevance of the evidence and consensus-based guidance on ACP content and process we provide. It should encourage healthcare professionals who care at least occasionally for persons with dementia, to conduct ACP with the person and family, and to continue when capacity of the person to take decisions diminishes. Such ACP fits with a person-centred, relational approach, which may be a prerequisite for ACP to manifest as truly beneficial.

With little specific high-quality evidence available, the Lancet dementia commission stated in 2017^10^ that “Whether advance care plans, made soon after the diagnosis of dementia, change outcomes or improve the quality of death is unknown.” We built upon an accumulating evidence base, and we found a consensus among experts from across the globe on when to offer initiating ACP: at or soon after diagnosis. With a public health approach, ACP can be offered before diagnosis, for example, to persons with MCI as shown in our model relating three specific issues in dementia. The relatively slow progress of dementia often provides opportunities for timely conversations. However, there was substantial ambiguity around addressing end-of-life issues at dementia diagnosis, which previously emerged as controversial.^28,29^ There are concerns about taking away hope and increasing anxiety. Others have reported that some families felt an information booklet about advanced dementia might increase anxiety,^30^ and in cancer patients, a question prompt list could increase anxiety in the short term but reduce it in the long term.^31^ We found a consensus that ACP conversations preferably also refer to the end of life, but it is not a requirement^14^ and, if addressed, to do so ‘a few months after diagnosis.’ Some people actually wish to talk about death and dying close to diagnosis, perhaps as a way of coping with the disease.^32^ It cannot be recommended generally as it may reinforce feelings of the diagnosis being a death sentence without any positive outlook. Sketching a realistic scenario of the future and its uncertainties, probing attitudes and offering choice, thus taking an individualised approach, may prevent possible detrimental effects of initiating ACP early on.

We achieved a consensus about repeating conversations and triggers for follow-up conversations. Ideally, a substantial number of 25 elements different in nature should be addressed in every conversation or at least once. Only five were regarded as optional, including care in the terminal phase. The same elements would apply in the case of young-onset dementia, while the scope of the issues addressed may be broader (e.g., address financial concerns) as also recommended by Flemish physicians.^33^

Among the optional elements of ACP was completing an advance directive (by the individual themselves), which Teno et al.^34^ already described in the 1990s as an optional constituent of the process of ACP. The elements along with suggestions when to address them, should not be seen as part of a delineated process. It offers a base, and additional tools that suggest content of care goals may be helpful. We did not prescribe topics or treatments to be addressed, such as social issues for persons with young-onset dementia or the withholding of particular medical treatments. Numerous studies have shown that people with dementia are at risk of overtreatment with burdensome medical interventions and undertreatment of symptoms, but also that the specific risks vary substantially by country and setting.^35–37^ For example, a national study in Sweden on dying with dementia showed that in nursing homes, pain and symptoms were more often assessed and treated than in hospitals, where over one-third (36.5%) received artificial ^fl^uids in the last 24 hours of life compared to 1.4% in nursing homes.^37^ In Israel, a person with advanced dementia who develops pneumonia likely receives artificial food and ^fl^uids (and antibiotics) after having been admitted to the hospital from home. In contrast, in the Netherlands, the person would be treated without artificial food and fluids-but possibly with oral antibiotics-in the nursing home, which is the residence of the large majority of people with advanced dementia, while probabilities in the US would be in-between the two countries.^36^ In addition, access to diagnosis, treatment, and care is not equitable; for example, it varied markedly between countries in the Western Pacific, from 568 (Brunei) to 360,869 (China) potential annual cases per service for dementia and cognitive decline assessment.^38^ In our Delph study, some experts from low-income and middle-income countries (LMICs) mentioned underdiagnosis of dementia related to limited access to memory services, which in turn, limits access to ACP.^20^ Topics and treatments to be addressed can be developed locally to fit with specific risks, needs, availability of care, and policy. Because a flexible approach may be even more important for persons with dementia and their family caregivers, and with little evidence for which method works better in which situation or with which persons, at present, we cannot recommend a particular scripted method.

Optimizing ACP in dementia is a complex and ambitious process that is usually best facilitated by multiple conversations over time. It requires substantial investment of all stakeholders with multiple elements addressed repeatedly. This raises the need to integrate its implementation as part of regular clinical practice in a culture of person-centred dementia care, healthcare management and policies. Healthcare professionals should guide the person with dementia and family and engage them in continued ACP conversations which need to become an embedded element of person-centred care organizational culture and practice. This requires time, which needs to be considered in terms of care management and available human resources. On the other hand, it may be reassuring that even untrained assistants can elicit meaningful responses from community-dwelling people with dementia through posing a few scripted open questions about what matters to them.^39^

### Limitations and Strengths

We did not select an exclusive panel with all having expertise in ACP in dementia care in clinical practice; most had an understanding of ACP or dementia care through research, a fifth (dementia care), or a quarter (ACP) through policy and administration activities. We believe the views of experts with either ACP or dementia care are important because they are among our target audience for providing and disseminating practice guidance. When asked at the conclusion of the study how the panel experienced participating in the Delphi study, between 52% and 30% of the panellist selected the options “a learning experience”, “relevant”, “difficult”, “worth the effort”, “rewarding to see improvement over rounds”, and/or “inspiring” while some experienced it as “burdensome” (16%), or “enjoyable” (5%). These responses are in line with our aim to avoid a low-risk approach to arrive at an immediate consensus without examining the exact controversy, which would have added little to existing knowledge. However, for feasibility, the study was still limited to four survey rounds, while a fifth round would have allowed for the examination of feasibility in practice or to prioritize the recommendations.

The professions of the experts were balanced as planned, with half of physicians^19^ and also 20% of nurses reflecting roles in ACP in the global literature (e.g., PubMed identifies ACP AND nurse about half of the hits of ACP AND physician). Indeed, in any model, whether a nurse-led, team-based, referral, or other, physician leadership and involvement are key.^40^ More than two-thirds of the panellists had private experience with advanced dementia at the end of life; we are unaware of evidence of the combined experience of professional and personal experience potentially limiting the broadness of their input or possibly missing perspectives unique to those with private experience only. Further, we involved young-onset dementia expertise and lived experience in a more inclusive approach, not limiting to dementia in old age as before.^24^ Asking persons with young-onset dementia directly how they wanted professional caregivers to approach ACP resulted in valuable responses and input in our Delphi study.

The recommendations integrate current knowledge from research, practice and lived experience; none of the recommendations have been reproduced unchanged from previous work. We did not use RAND/UCLA scaling and criteria for consensus which we found insufficiently conservative, overlooking rather than exposing ambiguities when applied post hoc in one of our previous Delphi studies.^41^ That is, a strength of our study is that we explored ambiguities in-depth over four survey rounds-while typically only two are planned-which offered ample opportunity for open-ended comments and resolution of ambiguities. The stepwise approach of developing a definitional framework from the first survey round offered an umbrella of three issues specific to ACP in dementia for clinical guidance on fluctuating capacity, involvement and role of family, and engagement and communication with the person with dementia. Our Delphi study would fall in the top 10% of Delphi studies reviewed by Diamond et al.,^42^ with at least 4 rounds, with over 100 panellists, and from multiple regions.

### Implications

The comprehensive set of recommendations clarifies a way forward in ACP in dementia. The individualized, holistic approach that is advocated implies that the specific guidance provided cannot be prescriptive. Rather, the many recommendations we provide highlight the need for a nuanced approach to addressing the highly personal and sensitive issues ACP is about. The advice of a Dutch person with dementia to clarify access to spiritual care to discuss existential issues judged as the top surprise (‘eye opening’) to the expert panel speaks to this. Person-centred communication can be learned as a skill and an attitude through interactive training that includes reflection and feedback.^43^ Communication skills may include observing behaviour and noting and responding to signs of distress.^13^

Professional caregivers may underestimate the capability of people with dementia themselves to talk about preferences related to ACP, and feel more comfortable talking with the family caregiver.^13^ However, in particular when initiated early, ACP potentially helps give voice to people with dementia to express their wishes for how to live with progressing illness and can help caregivers in addressing future care needs. Recent and ongoing research examines conversations in detail, which is promising in providing guidance on navigating dyadic conversations^44–47^ as guidance on how to communicate and engage persons with dementia and their families in ACP is particularly sparse. Future research should also increase an understanding of effective, flexible, and feasible pragmatic approaches that can be personalized, with policy initiatives supporting such approaches.^47^ A better understanding of what works for whom in the complex process of ACP requires adequately powered explanatory and pragmatic trials and other research designs. Further research is needed on experiences around raising the end of life, in particular focussing on community and hospital settings.

## Conclusion

We provide clinical recommendations that integrated findings from the literature with expert views. ACP in dementia surfaces as a highly complex and communicative person-centred practice, an ambitious process involving varying input of stakeholders over time. Personalizing of timing and contents is key. Pragmatic choices to tailor it to the person and family may be needed in practice also because our participants with lived experience desire coordinated care and conversations with a limited number of people involved. The guidance for healthcare professionals we provide can be useful in training programs and help healthcare professionals to reflect on their practice and further develop communication skills on-the-job. Policy and implementation initiatives need to carefully consider optimizing ACP vs. feasibility of good-enough ACP, and balance issues of inclusiveness and generalizability vs. where specific guidance is needed to optimize process and content for persons with dementia and their family.

## Supplementary Material

Supplemental B results

Supplemental A methods

Supplementary materials

Supplementary material associated with this article can be found in the online version at doi:10.1016/j.jpainsymman.2025.02.471.

## Figures and Tables

**Table 1:** The Three Issues Specific or of Particular Importance to ACP in Dementia: The Most Salient Recommendations and its Evaluations

Issue and Evaluation^a^	The Most Salient Recommendations (Primarily for Healthcare Providers^b^) That Achieved a Consensus^c^

**1) Capacity**^b^ ***Survey round 1** (*n = *91)*^a^ No consensus. Moderate agreement (median 4, IQR 1, 87.9% agreed). ***Survey round 3** (*n = *83)* (not included in round 2 to allow more time for revisions) Consensus on revised recommendations Very high agreement (median 5, IQR 0, 96.4% agreed)	• When starting an ACP conversation with a person with dementia,^b^ always start from the assumption that the person has capacity. • Formal capacity assessment is not necessary for every ACP conversation but should be performed if required guided by a country’s legal and regulatory frameworks. Nevertheless, it is important to be aware of any capacity issues occurring despite support in ACP conversations. If in doubt about a person’s capacity, short assessment tools can be used. • Keep in mind that capacity is decision-specific and may fluctuate over time: a person with dementia might have capacity for one decision and not for another, or their capacity may be better at a certain moment in time. Therefore, ACP conversations and its contents should be spread over time and planned in a flexible manner considering also triggers and opportunities to spontaneously start ACP conversations. *References:* Alzheimer Europe. Legal capacity and decision making: The ethical implications of lack of legal capacity on the lives of people with dementia. 2020. Harrison Dening K, Jones L, Sampson EL. Advance care planning for people with dementia: A review. Int Psychogeriatr. 2011;23:1535–1551. doi: 10.1017/S1041610211001608. Piers R, Albers G, Gilissen J, et al. Advance care planning in dementia: Recommendations for healthcare professionals. BMC Palliat Care. 2018 June 21;17(1):88. doi: 10.1186/s12904-018-0332-2. Wendrich-van Dael A, Bunn F, Lynch J, Pivodic L, Van den Block L, Goodman C. Advance care planning for people living with dementia: An umbrella review of effectiveness and experiences. Int J Nurs Stud. 2020;107:103576. doi: 10.1016/j.ijnurstu.2020.103576.

**2) Family**^b^ ***Survey round 1** (*n = *91)* *C*onsensus. High agreement (median 5, IQR 1, 93.4% agreed).	• Encourage family to listen to, discuss and appreciate the person’s deliberations and preferences, early (even prior to diagnosis), provided the person is willing to share this information. • Inform family about their changing role in the *ACP* process, including their (future) role to reconstruct or interpret the person’s probable preferences from current indications rather than to make their own decisions on the person’s behalf. • Prepare family for a nonlinear process of understanding the person’s preferences highlighting the possibility of contradictions between current understanding of preferences indicated verbally or nonverbally, and preferences stated earlier. *References:* Bruce CR, Bibler T, Childress AM, Stephens AL, Pena AM, Allen NG. Navigating ethical conflicts between advance directives and surrogate decision-makers’ interpretations of patient wishes. Chest. 2016 February;149(2):562–567. doi: 10.1378/chest.15-2209. Piers R, Albers G, Gilissen J, et al. Advance care planning in dementia: Recommendations for healthcare professionals. BMC Palliat Care. 2018 June 21;17(1):88. doi: 10.1186/s12904-018-0332-2.

**3) Engagement and communication** ***Survey round 1** (*n = *88)* Consensus. High agreement (median 5, IQR 1, 93.2% agreed)	**- Active role** • Conversations about preferences for future care must start early because the active role played in ACP inevitably reduces over time. At the very least, early agreement on who can represent the person with dementia and how much leeway the representative^b^ may have in interpreting these preferences. • Take time and start a conversation by making sure all agree about the purpose of the conversation (Groen van de Ven et al., 2017). Accept if the person does not want or cannot talk about future issues but return to this, as active role played may fluctuate and also readiness to engage in conversations may change. Also, another professional caregiver may return to it; a caregiver with a different-trust-base or no particular-relationship with the person with dementia or in a different setting such as at home (Karel et al., 2007; Goodman et al., 2013; Poppe et al., 2013; Tilburgs et al., 2018). • Making advance decisions or conveying rather confronting information is not advised if the person is not comfortable with that or copes by denying the diagnosis and not looking ahead (Thorsen et al., 2020). Discussing concrete everyday care experiences and key relationships on which the person with the dementia is the expert can normalize matters while it can help infer preferences about future care (Goodman et al., 2013; Poppe et al., 2013).
	**- Communication issues** • Address the person with dementia directly also when family is present as we tend to underestimate capacities to express preferences and let family take over (Godwin et al., 2009; Karnieli-Miller et al., 2012). • Express engagement and empathy such as through maintaining eye contact, gestures and intonation even when the person seems disengaged (Visser et al., 2021). Double check understanding. Purposefully use (Tilburgs et al., 2018) or not use (Goosens et al., 2020) closed questions to fit with person and culture, and reformulate or use images if needed. Offer opportunity to ask questions (Goossens et al., 2020). • Listen carefully to what the person is saying in an effort to also understand messages that are less clear, and let the person talk (Karel et al., 2007). Awareness of their body language, reading nonverbal behaviour is essential. Reading behaviour may provide a lot of information; specific types of dementia come with different behaviours. Always be mindful of sensitive topics as persons with dementia may have difficulties expressing feelings verbally, but also nonverbally. *References:* Godwin B. ‘In solitary confinement’: Planning end-of-life well-being with people with advanced dementia, their family and professional carers. Mortality 2009;14(3):265–285. doi: 10.1080/13576270903056840. Goodman C, Amador S, Elmore N, Machen I, Mathie E. Preferences and priorities for ongoing and end-of-life care: A qualitative study of older people with dementia resident in care homes. Int J Nurs Stud. 2013 December;50(12):1639–1647. doi: 10.1016/j.ijnurstu.2013.06.008. Goossens B, Sevenants A, Declercq A, Van Audenhove C. Improving shared decision-making in advance care planning: Implementation of a cluster randomized staff intervention in dementia care. Patient Educ Couns. 2020 April;103(4):839–847. doi: 10.1016/j.pec.2019.11.024. Groen van de Ven L, Smits C, Elwyn G, et al. Recognizing decision needs: First step for collaborative deliberation in dementia care networks. Patient Educ Couns. 2017 July;100(7):1329–1337. doi: 10.1016/j.pec.2017.01.024. Karel MJ, Moye J, Bank A, Azar AR. Three methods of assessing values for advance care planning: Comparing persons with and without dementia. J Aging Health. 2007 February;19(1):123–151. doi: 10.1177/0898264306296394. Karnieli-Miller O, Werner P, Neufeld-Kroszynski G, Eidelman S. Are you talking to me?! An exploration of the triadic physician-patient-companion communication within memory clinics encounters. Patient Educ Couns. 2012 September;88(3):381–390. doi: 10.1016/j.pec.2012.06.014. Poppe M, Burleigh S, Banerjee S. Qualitative evaluation of advanced care planning in early dementia (ACP-ED). PLoS One. 2013April 10;8(4):e60412. doi: . Thorsen K, Dourado MCN, Johannessen A. Awareness of dementia and coping to preserve quality of life: a five-year longitudinal narrative study. Int J Qual Stud Health Well-being. 2020 December;15 (1):1798711. doi: 10.1080/17482631.2020.1798711. Tilburgs B, Vernooij-Dassen M, Koopmans R, Weidema M, Perry M, Engels Y. The importance of trust-based relations and a holistic approach in advance care planning with people with dementia in primary care: A qualitative study. BMC Geriatr. 2018 August 16;18(1):184. doi: 10.1186/s12877-018-0872-6. Visser M, Smaling HJA, Parker D, van der Steen JT. How do we talk with people living with dementia about future care: A scoping review. Front Psychol. 2022 Apr 12;13:849100. doi: 10.3389/fpsyg.2022.849100 *[accepted manuscript; it was not available yet at the time we presented this text to the panel].*

a Evaluation criteria for consensus (defined as high or very high (dis)agreement): very high agreement, a median of 5 and an IQR of 0 and ≥80% scoring a 4 or 5; high agreement, a median 5 and an IQR ≤1 and ≥80% scoring a 4 or 5; moderate agreement, a median of 4–5 and an IQR ≤2 and ≥60% scoring a 4 or 5; low agreement, a median of 4–5, and an (IQR ≤ 2 or ≥ 60% scoring a 4 or 5); no agreement, a median 4–5 otherwise or a median >2 and <4. For consensus on disagreement, reverse median with the same IQR requirements and disagreement percentages (van der Steen etal., 2014).^24^

b Definitions provided to the panel: ***Capacity:*** meaning capacity to play a role in the ACP process which can include decision-making capacity or other capacity needed at some point, such as to communicate values; ***Persons with dementia:*** may have young-onset (YOD) or late-onset (LOD) dementia of any type and stage of dementia regardless of capacities; ***Family:*** the family caregiver, other family member, other relative or friend who knows and represents the patient and possible other family caregivers, family members, relatives or friends; therefore, encompassing more than biological or other family relationships (we referred to family in a broad sense; avoiding the term caregiver); ***Representative or proxy:*** can be the family but also an appointed legally authorized person who does not know the person with dementia well to serve as a proxy or substitute decision maker or advocate for the person with dementia when unable to decide him- or herself; ***Healthcare teams and healthcare providers:*** can include healthcare professionals and social care professions (this definition added after comments of the panel).

c Suppl B2 shows the phrasing of the interim recommendations that were revised during the process of achieving a consensus and the feedback provided to the panel.

Abbreviations: ACP = advance care planning; IQR = inter-quartile range.

**Table 2:** Recommendations on Personalizing ACP in Dementia

Final Statements That Achieved a Consensus^a^	Agreement

a. Healthcare professionals should adopt a person-centred approach when engaging in ACP conversations with the person with dementia and their family. This requires tailoring the ACP conversation to their health literacy, style of communication, and personal values, and to the person’s capacity for communication and decisions which may fluctuate (the round 3 statement which was included under a new heading ‘Recommended roles and tasks’ in round 3)	Very high agreement. Median 5, IQR 0, 98.8% agreed (*n* = 85)
**Adopt a prudent approach**	
b. ACP should be adapted to the individual’s capacity, understanding about ACP and readiness to engage in the ACP process of the person with dementia and the family (the round 1 statement)	Very high agreement. Median 5, IQR 0, 95.6% agreed (*n* = 91)
c. ACP includes exploring goals for future care but it may be helpful to start with discussing current care (the round 1 statement)	Very high agreement. Median 5, IQR 0, 95.6% agreed (*n* = 91)
d. In case there is some resistance or hesitance which risks ACP not happening before capacity of the person with dementia is substantially impaired, healthcare professionals should take time, gain trust, explore this resistance or hesitance and what elements of ACP the person would be ready for (e.g., exploring personal values or identifying a representative), and based on this, strongly encourage the ACP conversation and retry if needed (the round 3 statement, introduced in round 2 based on divergent comments in round 1, revised after round 2)^a^	High agreement. Median 5, IQR 1, 94.1% agreed (*n* = 85)
**Provide information**	
e. If desired by the patient or family after provided the opportunity to learn more, ACP includes information about diagnosis, probable disease course, and prognosis, and advantages and disadvantages of possible care and treatment options (the round 1 statement)	Very high agreement. Median 5, IQR 0, 95.6% agreed (*n* = 91)
f. Healthcare professionals should provide persons with dementia and their family with clear and coherent information concerning ACP and they should prioritize together with the person and the family what information is provided in case uptake of information is limited. Information on benefits and limitations of ACP must be provided as a minimum (the round 1 statement)	High agreement. Median 5, IQR 1, 87.9% agreed (*n* = 91)
**Explore understanding**	
g. The ACP process includes an exploration of the decisional capacity, understanding of ACP and an explanation of its aims, elements, benefits, limitations and legal status of the person with dementia and the representative (family) and an exploration of the relationship. Such explorations are repeated as necessary (the round 1 statement)	High agreement. Median 5, IQR 1, 94.4% agreed (*n* = 90)
h. ACP includes the exploration of understanding about the dementia and its course of the person with dementia and the family, and health-related experiences, concerns and personal values of the person with dementia across the physical, psychological, social and spiritual (the round 1 statement)	High agreement. Median 5, IQR 0.25, 94.4% agreed (*n* = 90)

a Supplement B3 shows the initial and revised statements and details the feedback of the panel. The criteria for consensus can be found in the Methods and in a footnote to Table 1.

**Table 3:** Recommendations on Timing of ACP

Recommendations That Achieved a Consensus^a^	Agreement

***Timing of initiation***	
a. Persons with mild cognitive impairment should be offered the opportunity to engage in ACP (the round 1 statement)	High agreement. Median 5, IQR 1, 84.8% agreed (*n* = 92)
b. Persons with dementia and family should be offered the opportunity to engage in ACP shortly after diagnosis^b^ (the round 1 statement)	Very high agreement. Median 5, IQR 0, 92.5% agreed (*n* = 93)
c. Persons with dementia and family should be offered the opportunity to engage in ACP at diagnosis^b^ (introduced in round 2; the round 2 statement)	High agreement. Median 5, IQR 1, 81.0% agreed (*n* = 84)
d. Anticipating progression of the disease, advance care planning is proactive. This implies it should start as soon as the diagnosis is made,^b^ when the patient can still be actively involved and patient preferences, values, needs and beliefs can be elicited (introduced in round 2; the round 2 statement)	High agreement. Median 5, IQR 1, 90.5% agreed (*n* = 84)
***Timing of ACP on end-of-life care relative to dementia diagnosis***	
e. ACP on end-of-life care should be tactfully introduced, explained and offered at a follow-up consultation a few months after disclosure of diagnosis^b^ (introduced in round 2, one of three alternatives presented in round 3)	High agreement. Median 5, IQR 1, 88.0% agreed (*n* = 83)
***Frequency of updating***	
f. As values and preferences may change over time, ACP conversations and documents should be updated at least yearly, and it may be more frequent as the clinical condition or personal situation changes (introduced in round 2; the round 2 statement)	High agreement. Median 5, IQR 1, 83.8% agreed (*n* = 80)
**Triggers for updating that achieved a consensus** ^c^	
***Triggers introduced in round 3 (n = 84)***	
● the person or family asking for palliative care	98.8%
● the person or family express information needs about prognosis or future	96.4%
● increased decline or increased fluctuation of health	92.9%
● rapidly declining capacity	91.7%
● care transition	91.7%
● undesirable emergency situations despite ACP	91.7%
***Triggers introduced in round 4 additionally suggested by the panel in round 3 (n = 89)***	
● perception that the APC plans do not adequately reflect the person’s preferences or values anymore	92.1%
● new services or treatment options become available and preferences may be discussed	89.9%
● rapidly declining ability of the person to communicate verbally	87.6%
● disagreement among family on goals of care or preferred treatment	85.4%
● concerns of the person or family about the process of ACP	84.3%

a Supplement B4 shows the initial and revised items and details the feedback of the panel, subsequent revisions and the process of achieving of consensus. It also shows three statements on ACP specifically on end-of-life care which did not achieve a consensus. The criteria for consensus can be found in the Methods and in a footnote to Table 1.

b Underlining was not presented to the panellists, but serves here to clarify the differences in referencing to diagnosis (some in different rounds).

c Triggers endorsed by at least 80% of the panel. See Supplement B4 for triggers that were endorsed by less than 80% of the panel.

**Table 4:** Advice From Persons With Young-Onset Dementia

Recommendation in Brief^a^	Surprise as Eye-Opening, % (n)	Most Valuable, % (n)

**1. Recommendations summarized from published results interviews persons young-onset dementia and family in Flanders** ^18^		
a. [Consider also nonmedical aspects]	2 (2)	55 (48)
b. [Discuss benefits for patient and family]	1 (1)	44 (38)
c. [Flexible approach guided by changes in person and family]	0 (0)	55 (48)
**2. Direct recommendations provided in interviews with community-dwelling persons with young-onset dementia in the Netherlands–most with member check of formulation Netherlands** ^25^		
a. Provide opportunities to discuss existential questions; refer to others if necessary	18 (16)	36 (31)
b. Don’t involve too many care providers without coordinating	14 (12)	41 (36)
c. Look closely at the person behind the dementia. Don’t judge based on the diagnosis alone	13 (11)	49 (43)
d. Limit the number of participants in the conversation. Provide the right conditions for everyone to have equal input	9 (8)	41 (36)
e. You, as a professional, are able to anticipate important decisions that need to be taken, so please be candid	9 (8)	36 (31)
f. Structure and prioritize discussion points so that the most important issues are dealt with first; the rest comes later	9 (8)	40 (35)
g. Ensure the environment is optimally conducive for the conversation with that person	7 (6)	39 (34)
h. When you focus on the dementia, do not forget to enquire after the basics	6 (5)	32 (28)
i. Focus on what the person wants	6 (5)	38 (33)
j. Schedule time to discuss uncomfortable topics	5 (4)	40 (35)
k. Listen carefully to really understand what someone needs	2 (2)	55 (48)

a Supplement B5 contains the recommendations, and for those obtained directly, it offers context which was also shown to the panel.

## Data Availability

Data supporting the study’s findings will be made available upon reasonable request with a proposal for additional analyses and after approval of the proposal from the taskforce chairs (JTvdS and IJK). Data collected for the analysis, including deidentified individual participant data and a data dictionary defining each field in the set including the consent item, will be made available upon granting such request. The study protocol which includes the planned analyses is publicly available at OSF.^19^

## Key Message

This Delphi study on advance care planning in dementia navigates ambiguities and derives clinical recommendations based on evidence along with consensus among experts from around the world. It provides a comprehensive set of best practice recommendations on optimal content and process for ACP for persons with dementia and their families.
