# Supplementary material for: Optimizing Advance Care Planning in Dementia: Recommendations From a 33-Country Delphi Study
Source: J Pain Symptom Manage. Author manuscript; Available in PMC 2025 Oct 24. (PMC12551429; doi:10.1016/j.jpainsymman.2025.02.471)
Supplement: Supplemental B results [file NIHMS2093260-supplement-Supplemental_B_results.docx]

**Supplement B. Results**

**Achieving a consensus on ACP in dementia:**

**Survey instrument and interim findings**

Supplemental material (2 of 2) with the article “Optimizing advance care planning in dementia: Recommendations from a 33-country Delphi Study”

1. Elements of ACP in dementia

2. Issues specific to ACP in dementia

3. Recommendations on personalizing elements of ACP in dementia

4. Recommendations on timing of initiating and updating ACP in dementia

5. Advice from persons with young-onset dementia

1. Elements of ACP in dementia

**Round 1** (text shown to the panel and analysed interim results; the final categories and selected findings are presented in the manuscript)

**Elements of Advance Care Planning (ACP) in dementia**

This section examines (1) what are the key elements of ACP in dementia, and (2) if there were multiple conversations what would you repeat each time. Acknowledging in practice this is often not the case, when rating elements of ACP in this round, we assume that both the person with dementia and family are involved and that multiple conversations are possible.

In this case, I feel that ACP conversations should comprise the following key elements:

| Possible elements (shown to the panel) | Response options shown to the panel and quantitative results round 1 | | | |
| --- | --- | --- | --- | --- |
|  | n | Yes, usually each time, % | Yes, usually once, % | No (please explain),^*^ % |
| 1. Assess concerns about current care and address these first or agree to discuss any such concerns in a separate conversation | 93 | 94 | 4 | 2 |
| 2. Explore the relationship between the person with dementia and their family | 93 | 63 | 35 | 1 |
| 3. Assess decisional capacity of the person with dementia | 91 | 90 | 7 | 3 |
| 4. Identify the representative role in future care and treatment decisions | 92 | 45 | 54 | 1 |
| 5. Discuss shared decision making (*note that this concerns both the person and family - definition of family repeated under "i" - both for all elements unless indicated otherwise*) | 90 | 69 | 29 | 2 |
| 6. Support both (persons with dementia and family) in their roles and tasks to share decision making | 89 | 96 | 4 | 0 |
| 7. Tailor the ACP conversation to the person’s capacity | 90 | 98 | 2 | 0 |
| 8. Inform the purpose of ACP | 90 | 57 | 42 | 1 |
| 9. Assess understanding of ACP | 89 | 67 | 33 | 0 |
| 10. Explore readiness to engage in ACP | 89 | 88 | 12 | 0 |
| 11. Tailor the ACP conversation to health literacy | 88 | 89 | 11 | 0 |
| 12. Tailor the ACP conversation to style of communication | 87 | 92 | 8 | 0 |
| 13. Tailor the ACP conversation to personal values | 87 | 87 | 11 | 1 |
| 14. Inform both (the person with dementia and their family) about the dementia and its course | 87 | 53 | 43 | 5 |
| 15. Explore understanding about the dementia and its clinical course | 85 | 62 | 36 | 1 |
| 16. If understanding about ACP or the dementia is limited and there is a risk of information overload, prioritize addressing information needs together | 86 | 87 | 13 | 0 |
| 17. Explore health-related experiences, concerns and personal values that would inform ACP | 86 | 58 | 42 | 0 |
| 18. Inform about global care goals (e.g. life prolongation, comfort) | 86 | 57 | 38 | 5 |
| 19. Decide on prioritized global care goal (e.g. life prolongation, comfort) | 86 | 70 | 26 | 5 |
| 20. Inform about possible specific care and treatment options | 85 | 81 | 16 | 2 |
| 21. Decide on specific future care and treatment | 85 | 68 | 22 | 9 |
| 22. Identify and discuss any global care goals and specific care and treatment that are not realistic | 87 | 67 | 24 | 9 |
| 23. Discuss and assess leeway for the representative to consider possible future care needs | 87 | 61 | 37 | 2 |
| 24. Complete an advance directive | 85 | 26 | 56 | 18 |
| 25. Agree on when to repeat ACP (set date for next conversation) | 86 | 66 | 15 | 19 |
| 26. Agree on when to repeat ACP (decide together on specific trigger events or experiences) | 86 | 72 | 24 | 3 |
| 27. Document any agreed upon global goals of care and preferences for specific future care and treatment | 87 | 84 | 14 | 2 |
| 28. Document a summary of the conversation, also in case no decisions are made | 87 | 92 | 7 | 1 |

^*^Explanations reported in this open-ended item comprise qualitative data that are available upon request.

29. Please use this space for any (brief) comments or suggestions

| (There were many comments about difficulty choosing because they miss nuances - they would favor a category in-between or a category “it depends” (on person, on circumstances)) |
| --- |

**Round 2** (main findings are highlighted in the manuscript)

**Elements of Advance Care Planning (ACP) in dementia**

**Summary of ratings in the first round**

All 28 proposed elements of ACP were endorsed by at least 80% of the panellists to conduct usually each time (most elements) or usually just once (a few elements). Support was lowest for “Complete an advance directive” (18% responded “no, not an element”) and agree on date when to repeat (19% responded “no, not an element”).

**This round**

We now ask you to rate whether you agree with the 3 categories below which we created based on the ratings.

**To what extent do you agree with the elements listed with CATEGORY 1 as a minimum to do at some point (if possible with the first conversation; it might be repeated but not necessarily so with each conversation)?**

Finding: consensus with high agreement (median 5, IQR 1, 91.7% agreed; n=84 excluding 1 do not know)


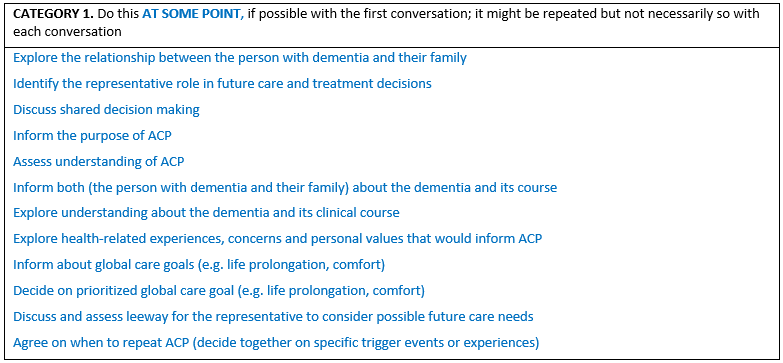


**In case you do not agree (moderately or strongly) with Category 1, which of the recommended elements would you revise or delete?**

Finding: 10 responses. Several panellists suggested to provide information about ACP before starting it, and to recommend the person and family talk together before starting ACP. Also, discussing global care goals may require multiple conversations or a next conversation, after exploring values. Addressing all these elements in one conversation would be too ambitious, asking for flexibility and valuing every single element addressed.

**In case you feel the list of elements with Category 1 should be expanded, please suggest the element that should be added?**

Finding: 10 responses, including one that it is comprehensive and clear. Again concerns about feasibility were expressed; if all are to be addressed in one conversation: the optimal may be the enemy of a good process. Further, establishing trust in the healthcare professional as a requirement to continue, and address legal issues depending on the jurisdiction.

**To what extent do you agree with the elements listed with CATEGORY 2 as a minimum to always do this, and repeat each conversation?**

Finding: consensus with high agreement (median 5, IQR 1, 88.2% agreed; n=85 excluding 1 do not know)


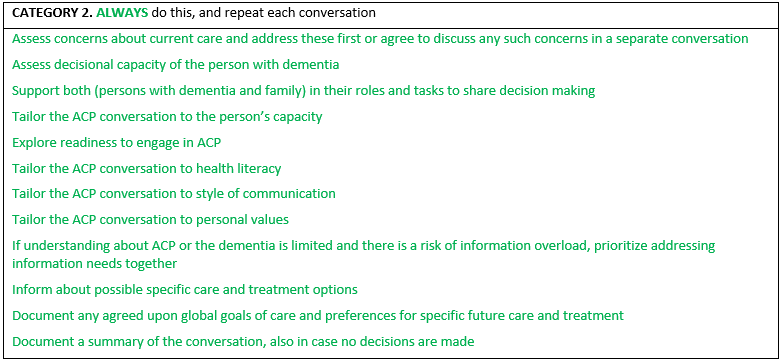


**In case you do not agree (moderately or strongly) with Category 2, which of the recommended elements would you revise or delete?**

Finding: 16 responses. Comments on Assessing decisional capacity went in different directions: to formalize it and not to formalize it. Again there were concerns about the elements being mandatory, critique referring to the term “always” as problematic to individualize conversations.

**In case you feel the list of elements with Category 2 should be expanded, please suggest the element that should be added?**

Finding: 6 responses. It included screening for caregiver distress, evaluating the conversation and asking the persons involved what they would like to address, and verifying if there have been any triggers or changes.

**To what extent do you agree with the elements listed with CATEGORY 3 as a minimum for optional elements - do this if it fits with the approach to ACP, context, situation, and legislation?**

Finding: consensus with high agreement (median 5, IQR 1, 92.7% agreed; n=82 excluding 1 do not know)


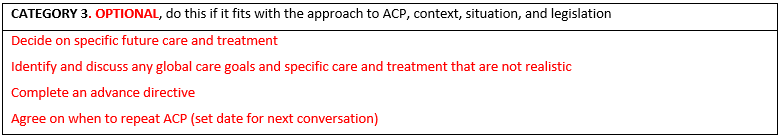


**In case you do not agree (moderately or strongly) with Category 3, which of the recommended elements would you revise or delete?**

Finding: 10 responses. It included healthcare professional’s interpretation as unrealistic which may also be seen as dealing with hope. Comments on completing an advance directive were to at least discuss the advantages or stimulate it. Some preferred setting a date for the next conversation in a non-optional category.

**In case you feel the list of elements with Category 3 should be expanded, please suggest the element that should be added?**

Finding: 5 responses. There were suggestions to move to other categories, and to specify (for particular care, and add examples), and a suggestion to assess what is currently important to the person that is relevant to future decisions about care.

**To what extent do you agree that it is feasible in most cases to conduct a series of ACP conversations and cover all elements of Categories 1 and 2?**

Finding: no consensus with moderate agreement (median 4, IQR 1, 72% agreed; n=79 excluding 3 do not know)

**On average, how many ACP conversations do you believe are needed with a person with dementia (in case family attends as well) to cover the elements of Categories 1 and 2? (0-30)**

Finding: median 4, IQR 3 (running from 3 to 6), range 0 (2 responses) to 15 (1 response); mean 4.80 (SD 3.052), n= 79

**To what extent do you agree that the elements of ACP should be the same for persons with young-onset dementia?**

Finding: Consensus with high agreement (median 5, IQR 1, 88.9% agreement, n=81 (excluding 2 do not know)

Panellists with young-onset dementia expertise: Consensus with high agreement (median 5, IQR 1, 93.3% agreement, n=15

**In case you do not agree (moderately or strongly), which of the elements would you change for persons with young-onset dementia?**

Finding: 14 responses. Most mentioned more social issues needed to be addressed such as children, finances. Some mentioned less medical issues due to fewer comorbidities, and that it is “often much more difficult,” a need for “more regular conversations,” yet “treatments and preferences may differ but the process should be similar.”

**To what extent do you agree that the elements of ACP should be the same for persons with mild cognitive impairment?**

Finding: No consensus with moderate agreement (median 4, IQR 1, 84.0% agreement, n=81 (excluding 3 do not know)

**In case you do not agree (moderately or strongly), which of the elements would you change for persons with mild cognitive impairment?**

Finding: 16 responses. Most commented on a more uncertain prognosis and therefore education about the course could be about prevention of decline and co-morbid disease, and dementia-related problems and treatments such as artificial nutrition and hydration would not be discussed.

**To what extent do you agree that the elements of ACP should be the same for persons with no dementia?**

Finding: No consensus with moderate agreement (median 4, IQR 2, 70.0% agreement, n=80 (excluding 2 do not know)

**In case you do not agree (moderately or strongly), which of the elements would you change for persons with no dementia?**

Finding: 27 responses. Most commented on capacity, and some on family involvement which may not be necessary. Again dementia-specific content and education would not be needed, “but the items addressed in the categories are excellent areas for consideration for anyone looking to engage in ACP.”

There were also questions if this concerned a person with some other disease than dementia (and this depending on the disease) or no disease.

**To what extent do you agree that a 4th Category is needed for ACP conversations specific to the terminal phase, referring to, for example, a person’s and family’s detailed preferences regarding rituals, funeral arrangements, and bereavement care?**

Finding: No consensus with moderate agreement (median 4, IQR 1, 76.3% agreement, n=80 (excluding 3 do not know))

**Please use this space for any (brief) comments or suggestions**

Part of the comments were similar to the comments provided in the open-ended items above. Regarding a 4^th^ category, some commented on elements such as funeral arrangements drifting away from medical domain, and a chaplain to help with discussions about rituals, funerals etc. Some people have strong views on funerals however, and it might be a good entry point but for others it may deter from having conversations, so this is very individual. Others commented that ACP sometimes only starts in the terminal phase and it could be appropriate to address. Such 4^th^ category could be broadened to fulfill last wishes.

**Round 3** (analysed interim results; highlighted findings in the manuscript)

**Summary of ratings in the second round**

The three categories with elements of ACP (“do at some point” “always do this” and “optional” all achieved a consensus (median score 5). Together, we developed a clear basis! However, the panel did not achieve a consensus (median 4) on whether a fourth category is needed for ACP conversations that specifically address the terminal phase. Reviewing the valuable feedback provided, we recognize that the three categories at this point all recommend about what to address in general terms, with no detail about the contents such as what specific treatments could be discussed. As the panel did agree earlier on that addressing end-of-life issues is not necessary for ACP in dementia, we suggest to add discussion of terminal care to the third, “OPTIONAL” category.

**To what extent do you agree that to Category 3 below (“OPTIONAL”) “Discuss preferences for care and rituals in the terminal phase” should be added?**

Finding: consensus with high agreement, median 5, IQR 1, 84.6% agreement, n=78 (excluding 5 don’t know))

**Please use this space for any (brief) comments or suggestions**

Finding: 13 responses. Some stated they agreed with optional because it depends on cultural and religious beliefs. Some commented to not understand what is meant by “optional” and on the terms used - living will as used for a legal document in some countries and disliking the term rituals used in category 3.

Completing an advance care directive (living will) is included among the optional elements of ACP, but it may have been completed but not brought to the attention of the healthcare professional. Please rate below to what extent you agree that the healthcare professional should verify whether there is any.

**To what extent do you agree that to Category 1 below , (“do this AT SOME POINT”), “Verify whether the person with dementia has any previous wishes in writing and check correct understanding” should be added?**

Finding: Consensus with high agreement (median 5, IQR 1, 96.1% agreement, n=76 (excluding 3 don’t know))

The elements of ACP had been abstracted from earlier work and adapted for dementia and we expected the set to be complete. Yet, we received an interesting suggestion about reflecting on the relationship with the healthcare professional, and we ask you to rate if the two elements below may indeed fill a gap in Category 2, (“ALWAYS do this”).

**To what extent do you agree that to Category 2 below (“ALWAYS do this”) “****Invest in building a trustful relationship with the person and family” should be added?**

Finding: Consensus with high agreement (median 5, IQR 1, 94.0% agreement, n=67 (excluding 3 don’t know))

**To what extent do you agree that to Category 2 above (“ALWAYS do this”) “Assess to what extent the person and family feel free to disagree with you as a healthcare professional, or bring up their own issues” should be added?**

Finding: No consensus with moderate agreement (median 4, IQR 2, 71.6% agreement, n=74 (excluding 4 don’t know))

**Please use this space for any (brief) comments or suggestions**

Finding: 18 responses. There were concerns about verifying wishes in writing leading to pressure in ACP conversations. Regarding investing in a trustful relationship, this would go without saying, or they are implicit or standards of professional practice and should therefore not be included in a list of elements. Another commented that the two proposed “always do this” additions set up the healthcare professional for failure, too aspirational and resulting in ACP not happening at all. Regarding assessing whether person and family feel free, there were a number of comments. One positive comments was about understanding conflicts. Other comments were negative: it was perceived as inappropriately assessing one’s own skills or part of overall communication skills which therefore need not be assessed. It was also unclear to some (“disagree on what?”). Others commented this just goes too far, how to assess it and also that it would be unclear what to do with the answer.

There was a consensus that the elements of ACP equally apply to persons with young-onset dementia (median 5), but not so for persons with no dementia (median 4 for MCI, and for other diseases) with reasons including that capacity, family involvement, and communication differ and that the clinical course for MCI is even more uncertain. Feasibility of addressing the long lists of elements is an issue (median rating 4 for feasibility, no consensus), and some commented this to be even more of an issue for persons with young-onset dementia.

**To what extent do you agree that, generally, the process of ACP in the case of young-onset dementia is more difficult due to more social issues involved (such as employment and raising a young family) than with ACP in older persons with dementia?**

Finding: No consensus with moderate agreement (median 5, IQR 1, 77.3% agreement, n=66 (excluding 7 do not know))

Panellists with young-onset dementia expertise: No consensus with no agreement (median 4, IQR 2.5, 69.2% agreement, n = 13).

**Please use this space for any (brief) comments or suggestions**

Finding: 13 responses. Some commented that expectations or other issues such as behavioral issues may make ACP more difficult, but also more important in the case of persons with young-onset dementia. More commented on difficulty depending on the individual or on many factors which are not so much related to different content of ACP with YOD (e.g., “I think there may be more/different issues but not that this makes ACP more difficult.” Also more commented on in essence, ACP process or also content being the same, some referring to person-centeredness as well: “it is not a question of age, but a question of exploring wat it is meaningful for the person at this time” “The process of ACP is the same: tailored to the specific situation. The issues at stake are not only social issues, but relate to all aspects of life.” Also, “best to keep focus on person-centre care and the needs of the person, rather than make assumptions based on diagnosis.” Some also commented on little evidence being available (e.g., “suggest there should be a disclaimer recognising how little is known about end of life care needs and experiences for this group.”

2. Issues specific to ACP in dementia

(Supplement 2 reports survey content as shown to the panel; the evaluations are in the manuscript)

**Survey round 1 – Capacity recommendations shown to the panel**

**The text below provides the most salient recommendations about capacity in relation to *ACP* in dementia**

1: strongly disagree

2: moderately disagree

3: neither agree nor disagree

4: moderately agree

5: strongly agree

Don't know

| If in doubt about a person’s capacity, assess using minimally invasive tools and observations. Knowing the person will help to facilitate the best outcome by optimizing their ability to understand and participate in *ACP* by, for example, avoiding distractions. A capacity assessment should be verified or repeated as necessary (*Peisah et al., 2018; Stuart & Thielke, 2018*).  Care should be taken to avoid undue or unclear influence of others when integrating shared decision-making principles to support decision-making capacity (*Harrison Dening et al., 2013; Scholten & Gather, 2018*).  *(View “i” under item number 3 for references)*  *References:*  Harrison Dening K, Jones L, Sampson EL. Preferences for end-of-life care: A nominal group study of people with dementia and their family carers. Palliat Med. 2013 May;27(5):409-417. doi: 10.1177/0269216312464094  Peisah C, Sorinmade OA, Mitchell L, Hertogh CMPM. Decisional capacity: Toward an inclusionary approach. Int Psychogeriatr. 2013 October;25(10):1571-1579. doi: 10.1017/S1041610213001014  Scholten M, Gather J. Adverse consequences of article 12 of the UN Convention on the Rights of Persons with Disabilities for persons with mental disabilities and an alternative way forward. J Med Ethics. 2018 April;44(4):226-233. doi: 10.1136/medethics-2017-10441  Stuart RB, Thielke S. Protocol for the assessment of patient capacity to make end-of-life treatment decisions. J Am Med Dir Assoc. 2018 February;19(2):106-109. doi: 0.1016/j.jamda.2017.11.011 |
| --- |

**Please use this space for any (brief) comments or suggestions**

|  |
| --- |

**Survey round 1 – Family recommendations shown to the panel**

**The text below provides the most salient recommendations about family in relation to** *ACP* **in dementia**

1: strongly disagree

2: moderately disagree

3: neither agree nor disagree

4: moderately agree

5: strongly agree

Don't know

| Encourage family to listen to, discuss and appreciate the person’s deliberations and preferences, early (even prior to diagnosis) provided the person is willing to share this information.  Inform family about their changing role in the *ACP* process, including their (future) role to reconstruct or interpret the person’s probable preferences from current indications rather than to make their own decisions on the person’s behalf.  Prepare family for a non-linear process of understanding the person’s preferences highlighting the possibility of contradictions between current understanding of preferences indicated verbally or non-verbally, and preferences stated earlier.  *(View “i” under item number 3 for references)*  *References:*  Bruce CR, Bibler T, Childress AM, Stephens AL, Pena AM, Allen NG. Navigating ethical conflicts between advance directives and surrogate decision-makers' interpretations of patient wishes. Chest. 2016 February;149(2):562-567. doi: 10.1378/chest.15-2209  Piers R, Albers G, Gilissen J, De Lepeleire J, Steyaert J, Van Mechelen W, Steeman E, Dillen L, Vanden Berghe P, Van den Block L. Advance care planning in dementia: Recommendations for healthcare professionals. BMC Palliat Care. 2018 June 21;17(1):88. doi: 10.1186/s12904-018-0332-2 |
| --- |

**Please use this space for any (brief) comments or suggestions**

|  |
| --- |

**Survey round 1 – Engagement and communication recommendations shown to the panel**

**The text below provides the most salient recommendations about engagement and communication in relation to** *ACP* **in dementia**

1: strongly disagree

2: moderately disagree

3: neither agree nor disagree

4: moderately agree

5: strongly agree

Don't know

| *Engagement and communication*  *- Active role*  Conversations about preferences for future care must start early because the active role played in *ACP* inevitably reduces over time. At the very least, early agreement on who can represent the person with dementia and how much leeway the representative may have in interpreting these preferences.  Take time and start a conversation by making sure all agree about the purpose of the conversation (*Groen van de Ven, et al., 2017*). Accept if the person does not want or cannot talk about future issues but return to this, as active role played may fluctuate and also readiness to engage in conversations may change. Also, another professional caregiver may return to it; a caregiver with a different-trust-base or no particular-relationship with the person with dementia or in a different setting such as at home (*Karel et al., 2007; Goodman et al., 2013; Poppe et al., 2013; Tilburgs et al., 2018*).  Making advance decisions or conveying rather confronting information is not advised if the person is not comfortable with that or copes by denying the diagnosis and not looking ahead (*Thorsen et al., 2020*). Discussing concrete everyday care experiences and key relationships on which the person with the dementia is the expert can normalize matters while it can help infer preferences about future care (*Goodman et al., 2013; Poppe et al., 2013*).  *- Communication issues*  Address the person with dementia directly also when family is present as we tend to underestimate capacities to express preferences and let family take over (*Godwin 2009; Karnieli-Miller et al., 2012*).  Express engagement and empathy such as through maintaining eye contact, gestures and intonation even when the person seems disengaged (*Visser et al., 2021*). Double check understanding. Purposefully use (*Tilburgs et al., 2018*) or not use (*Goosens et al., 2020*) closed questions to fit with person and culture, and reformulate or use images if needed. Offer opportunity to ask questions (*Goossens et al., 2020*).  Listen carefully to what the person is saying in an effort to also understand messages that are less clear, and let the person talk (*Karel et al., 2007*). Awareness of their body language, reading nonverbal behaviour is essential. Reading behaviour may provide a lot of information; specific types of dementia come with different behaviours. Always be mindful of sensitive topics as persons with dementia may have difficulties expressing feelings verbally, but also nonverbally.  Godwin B. ‘In solitary confinement’: Planning end-of-life well-being with people with advanced dementia, their family and professional carers. Mortality 2009;14(3):265-285. DOI: 10.1080/13576270903056840  *(View “i” under item number 3 for references)*  Goodman C, Amador S, Elmore N, Machen I, Mathie E. Preferences and priorities for ongoing and end-of-life care: A qualitative study of older people with dementia resident in care homes. Int J Nurs Stud. 2013 December;50(12):1639-1647. doi: 10.1016/j.ijnurstu.2013.06.008  Goossens B, Sevenants A, Declercq A, Van Audenhove C. Improving shared decision-making in advance care planning: Implementation of a cluster randomized staff intervention in dementia care. Patient Educ Couns. 2020 April;103(4):839-847. doi: 10.1016/j.pec.2019.11.024  Groen van de Ven L, Smits C, Elwyn G, Span M, Jukema J, Eefsting J, Vernooij-Dassen M. Recognizing decision needs: First step for collaborative deliberation in dementia care networks. Patient Educ Couns. 2017 July;100(7):1329-1337. doi: 10.1016/j.pec.2017.01.024  Karel MJ, Moye J, Bank A, Azar AR. Three methods of assessing values for advance care planning: Comparing persons with and without dementia. J Aging Health. 2007 February;19(1):123-151. doi: 10.1177/0898264306296394  Karnieli-Miller O, Werner P, Neufeld-Kroszynski G, Eidelman S. Are you talking to me?! An exploration of the triadic physician-patient-companion communication within memory clinics encounters. Patient Educ Couns. 2012 September;88(3):381-390. doi: 10.1016/j.pec.2012.06.014  Poppe M, Burleigh S, Banerjee S. Qualitative evaluation of advanced care planning in early dementia (ACP-ED). PLoS One. 2013 April 10;8(4):e60412. doi: 10.1371/journal.pone.0060412  Thorsen K, Dourado MCN, Johannessen A. Awareness of dementia and coping to preserve quality of life: a five-year longitudinal narrative study. Int J Qual Stud Health Well-being. 2020 December;15(1):1798711. doi: 10.1080/17482631.2020.1798711.  Tilburgs B, Vernooij-Dassen M, Koopmans R, Weidema M, Perry M, Engels Y. The importance of trust-based relations and a holistic approach in advance care planning with people with dementia in primary care: A qualitative study. BMC Geriatr. 2018 August 16;18(1):184. doi: 10.1186/s12877-018-0872-6  Visser M, Smaling HJA, Parker D, van der Steen JT. How do we talk with people living with dementia about future care: A scoping review. Manuscript with accepted abstract for special issue, 2021 |
| --- |

**Please use this space for any (brief) comments or suggestions**

|  |
| --- |

**Survey round 3 – Capacity recommendations and feedback shown to the panel**

**(the three issues were not included in survey round 2)**

**Original statement and text**:

2. The text below provides the most salient recommendations about capacity in relation to ACP in dementia

| If in doubt about a person’s capacity, assess using minimally invasive tools and observations. Knowing the person will help to facilitate the best outcome by optimizing their ability to understand and participate in *ACP* by, for example, avoiding distractions. A capacity assessment should be verified or repeated as necessary (*Peisah et al., 2018; Stuart & Thielke, 2018*).  Care should be taken to avoid undue or unclear influence of others when integrating shared decision-making principles to support decision-making capacity (*Harrison Dening et al., 2013; Scholten & Gather, 2018*).  *References:*  Harrison Dening K, Jones L, Sampson EL. Preferences for end-of-life care: A nominal group study of people with dementia and their family carers. Palliat Med. 2013 May;27(5):409-417. doi: 10.1177/0269216312464094  Peisah C, Sorinmade OA, Mitchell L, Hertogh CMPM. Decisional capacity: Toward an inclusionary approach. Int Psychogeriatr. 2013 October;25(10):1571-1579. doi: 10.1017/S1041610213001014  Scholten M, Gather J. Adverse consequences of article 12 of the UN Convention on the Rights of Persons with Disabilities for persons with mental disabilities and an alternative way forward. J Med Ethics. 2018 April;44(4):226-233. doi: 10.1136/medethics-2017-10441  Stuart RB, Thielke S. Protocol for the assessment of patient capacity to make end-of-life treatment decisions. J Am Med Dir Assoc. 2018 February;19(2):106-109. doi: 0.1016/j.jamda.2017.11.011 |
| --- |

**Summary of comments to original statement:**

The panel provided a wide range of important feedback to the two original recommendations. These included the need to simplify language (many found formulations too complex), the need to consider that capacity depends upon the context (e.g. influence of environment, what moment in the day) and subject (e.g. people can say what it of value to them even if they cannot choose a specific preferred treatment at the end of life), that capacity fluctuations over time which impacts what can be discussed when, that tools are not always necessary but observation of capacity during every conversation is important, that legal requirements will differ per country, adequate training needed in evaluating capacity, and considerations of when to revisit. The idea of undue influence was not clear.

Further, the panel commented on communication and the importance of supported decision-making to enhance capacity, and the relational component of ACP (e.g. that family have an influence could be negative or positive). We will consider these suggestions when writing about Engagement and communication (the third issue specific for dementia [note this refers to the summary, reference 12 in the article]). Based on the comments on the capacity recommendations and an additional screening of the literature on capacity, ACP and decision-making, we have redrafted the recommendations.

**2. Revised recommendations:**

| When starting an ACP conversation with a person with dementia, always start from the assumption that the person has capacity  Formal capacity assessment is not necessary for every ACP conversation but should be performed if required guided by a country’s legal and regulatory frameworks. Nevertheless, it is important to be aware of any capacity issues occurring despite support in ACP conversations. If in doubt about a person’s capacity, short assessment tools can be used.  Keep in mind that capacity is decision-specific and may fluctuate over time: a person with dementia might have capacity for one decision and not for another, or their capacity may be better at a certain moment in time. Therefore, ACP conversations and its contents should be spread over time and planned in a flexible manner considering also triggers and opportunities to spontaneously start ACP conversations.  *References:*  Alzheimer Europe. Legal capacity and decision making: The ethical implications of lack of legal capacity on the lives of people with dementia. 2020.  Harrison Dening K, Jones L, Sampson EL.. Advance care planning for people with dementia: A review. Int Psychogeriatr. 2011;23:1535–1551. doi: 10.1017/S1041610211001608  Piers R, Albers G, Gilissen J, De Lepeleire J, Steyaert J, Van Mechelen W, Steeman E, Dillen L, Vanden Berghe P, Van den Block L. Advance care planning in dementia: Recommendations for healthcare professionals. BMC Palliat Care. 2018 June 21;17(1):88. doi: 10.1186/s12904-018-0332-2.  Wendrich-van Dael A, Bunn F, Lynch J, Pivodic L, Van den Block L, Goodman C. Advance care planning for people living with dementia: An umbrella review of effectiveness and experiences. Int J Nurs Stud. 2020;107:103576. doi: 10.1016/j.ijnurstu.2020.103576 |
| --- |

**Revised statement:**

2. The **revised text above** provides the most salient recommendations about capacity in relation to ACP in dementia

**Panel rating of original statement:**

Median 4 but no consensus

**Your level of agreement with original statement**: please refer to your email

**2 Please rate your level of agreement with the revised statement**

1: strongly disagree

2: moderately disagree

3: neither agree nor disagree

4: moderately agree

5: strongly agree

Don't know

**3. Please use this space for any other (brief) comments or suggestions**

|  |
| --- |

**Survey round 4 - Feedback to the panel only**

We presented three issues specific to ACP in dementia (capacity, family, and engagement & communication). We visualized its relationships in a Figure which we refined with your help in three iterations, until all of this achieved a consensus! We get back to you with the three issues in section E, on research priorities.

3. Recommendations on personalizing ACP in dementia

(text shown to the panel and the findings on statement d in Table 2 that did not achieve a consensus immediately; the final statements that achieved a consensus over 3 rounds are presented in the manuscript)

**Round 1**

The first round included a section “Selected recommendations regarding Advance Care Planning (ACP) in dementia” with subheadings: Timing, Providing Information, Exploring Understanding, and Prudent Approach.

**Round 2**

Section Selected recommendations regarding ACP in dementia

**Prudent approaches**

As with the recommendations on timing, recommendations on providing information, exploring understanding and a prudent approach all achieved a consensus (median 5 for the 6 items, 2 each).

We received interesting comments on prudent approaches. Many advocated a careful approach, saying for example, we “should not impose,” or warning against “too much as a must” and against an overwhelming amount of information. However, others felt we need to push a bit to discuss before capacity loss, and to not spend too much time on consent to participate *ACP* in a population with limited reserve who may fatigue easily.

Please rate the following new statement.

**In case there is some resistance or hesitance which risks *ACP* not happening before capacity of the person with dementia is substantially impaired, healthcare professionals should strongly encourage the *ACP* conversation**

Finding: No consensus with moderate agreement (median 4, IQR 2, 69.3% agreement, n=75 excluding 1 don’t know)

**Please use this space for any (brief) comments or suggestions**

Finding: 34 responses. “Strongly encourage” was considered by many as too forceful, too proactive, paternalistic, or as mandating, coercion, pressure or pushing too hard. Treating people with respect and a person-centred approach would not allow going beyond “insisting” or “encouraging.” This is because people should be ready for an ACP conversation, open to it, or in the right stage of behavioural change. Otherwise, an overly proactive approach may not gain trust, lead to fear that people entered a terminal phase, or to rushed decisions that family may then feel bound to uphold. There was also a comment that it may be wise to accept but also to explore why there was resistance of hesitance, and healthcare professionals need to be trained in dealing with emotional reactions and defense mechanisms. Further, the urgency was emphasized in this being the only chance to protect a person’s right to autonomy, while others emphasized the right to not be involved in ACP.

**Round 3**

Section Selected recommendations regarding ACP in dementia

**Timing - Prudent approaches**

As we had received opposing comments from the panel on a careful approach and accepting hesitance to engage in *ACP* versus a need to push a bit, we expected the new statement about strong encouragement in the case of hesitance or resistance to be controversial. Again, we could incorporate the panel’s critiques and suggestions in revising the statement and adding what the healthcare provider should also do when insisting on or encouraging *ACP*.

[
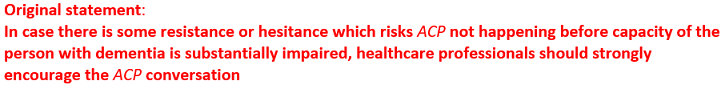
](https://data.castoredc.com/view-file/6909E0E4-8F2B-4282-B273-63154B497DF1/image/544)

**Panel rating of original statement:**

*Median 4*, no consensus

**Your level of agreement with original statement:** please refer to your email (**C7 prudent approaches**)

[
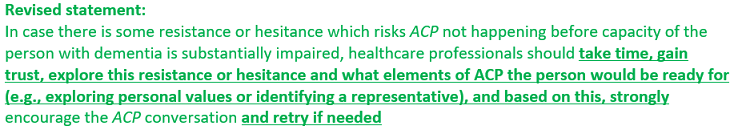
](https://data.castoredc.com/view-file/6909E0E4-8F2B-4282-B273-63154B497DF1/image/542)

**Please rate your level of agreement with the revised statement above:**

Finding: Consensus with high agreement (median 5, IQR 1, 94.1% agreement, n=85)

**Please use this space for any (brief) comments or suggestions**

Finding: 33 responses. Comments referred to the preference of the person whether to engage in ACP should be decisive, and self-determination be applied to conducting ACP. The panellists generally liked the additions and some stated the revisions improved matters as conveying the sensitivities and a more person-centred approach and found it well-formulated. However, others suggested to rephrase, for example, to split up in more sentences or to shorten. There were still objections to the term “strongly” as it feels imperative or invasive. Some panelist would not retry a (full) ACP conversation, but make sure a proxy decision maker is identified, or would just talk about “values and preferences.”

4. Recommendations on timing of initiating and updating ACP in dementia

(text shown to the panel and recommendations that did not achieve a consensus; the recommendations that achieved a consensus are presented in the manuscript)

**Round 1**

**Section Selected recommendations regarding**ACP**in dementia: Timing**

**(The statements that immediately achieved a consensus in round 1 are all in the manuscript (Table 3, a and b))**

**Round 2**

**Section Selected recommendations regarding**ACP**in dementia: Timing**

The panel agreed upon the two statements in the form of recommendations in the previous round that addressed timing (consensus, median 5):

“Persons with mild cognitive impairment should be offered the opportunity to engage in ACP” and

“Persons with dementia and family should be offered the opportunity to engage in ACP shortly after diagnosis.”

This round, we ask you to also rate other recommendations in the domain of timing. Note that there are small, but potentially important differences with the first three of the following statements.

**(The statements that immediately achieved a consensus in round 2 are also in the manuscript (Table 3, c and d))**

**ACP on end-of-life care should be initiated at the time of diagnosis of dementia**

Finding: No consensus with no agreement (median 3, IQR 2, 45.2% agreement, n=84 excluding 1 don’t know).

**Please use this space for any (brief) comments or suggestions**

Finding: 50 responses commenting on the above statement and the two statements that were not on end of life that achieved a consensus. “Offering” was found less “intrusive” than starting “as soon as possible.”

Regarding timing of bringing up the end of life, the panellists called for a person-centred approach while at diagnosis may be too early for some. For example, “might be too overwhelming” “might not be aligned with needs” or “readiness” The panellists suggested to defer bringing up end of life, asking if they are ready for that, to a follow-up conversation for example 4 to 6 weeks after diagnostic disclosure in order to ***first***: allow time to come to deal with immediate issues, come to terms with the diagnosis, time to digest, the information to settle bit, to give information about the progression of the illness, to explain the course of the disease and its implications, to allow time to understand the condition, to get a realistic view of what living with the condition means, to advice on what to do to help preserve quality of life, to give post-diagnostic support, to build trust, to avoid a conversation when people are in anger or denial. There were concerns that bringing up the end of life at diagnosis will “cause emotional harm”, “cause anxiety and depression”, be “frightening”, “blunt” “nihilistic”, “imply that life is over” “add to stigma”, risk “miss out on quality-of-life-enhancing treatment” and “increase resistance and hesitancy.”

**Round 3**

**Section Selected recommendations regarding**ACP**in dementia: Timing - Initiation**

The panel agreed upon offering *ACP* at diagnosis (consensus, median 5), but was very divided (no consensus, median 3) about whether this should include conversations on care at the end of life.

We indeed expected this to be controversial and, we are grateful for the panel’s extensive feedback. We incorporated the feedback by revising the statement in three ways, adding panelists’ various suggestions about situations in which addressing end-of-life care could be appropriate.

[
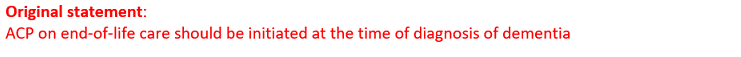
](https://data.castoredc.com/view-file/6909E0E4-8F2B-4282-B273-63154B497DF1/image/346)

**Panel rating of original statement:**

*Median 3*, no consensus

**Your level of agreement with original statement:** please refer to your email (**C3 initiation**)

**Three revised statements:**

[
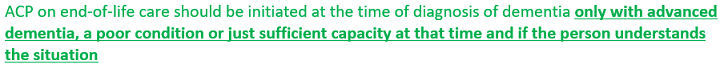
](https://data.castoredc.com/view-file/6909E0E4-8F2B-4282-B273-63154B497DF1/image/536)

**Your level of agreement with the revised statement above:**

Finding: No consensus with no agreement (median 3.5, IQR 3, 50.0% agreement, n=84 excluding 1 don’t know).


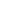


[
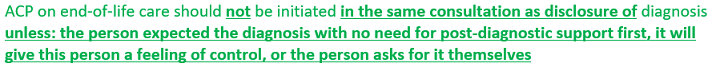
](https://data.castoredc.com/view-file/6909E0E4-8F2B-4282-B273-63154B497DF1/image/538)

**Your level of agreement with the revised statement above:**

Finding: No consensus with moderate agreement (median 4, IQR 1, 77.1% agreement, n=83 excluding 1 don’t know).

[
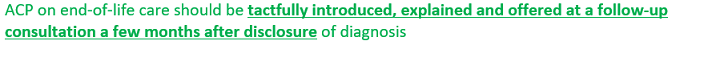
](https://data.castoredc.com/view-file/6909E0E4-8F2B-4282-B273-63154B497DF1/image/540)

**Your level of agreement with the revised statement above:**

Finding (statement e in Table 3 in the manuscript): Consensus with high agreement (median 5, IQR 1, 88.0% agreed (n=83)

**Please use this space for any (brief) comments or suggestions**

Finding: 44 responses. The urgency for an opportunity to discuss the end of life expressed with “just sufficient capacity” was not understood, and the wording of the first two statements was repeatedly commented on as difficult to follow (“feel like too much is being done to squeeze in all of the multiple comments.”) Some people would expect the diagnosis yet would still be shocked to hear it. Post-diagnostic support is needed, and the people who feel no need, may in fact need it the most.

The usefulness of recommending to introduce tactfully was questioned as professionals may be inclined to do it not explicitly enough, and “dementia is a terminal condition. To avoid this reality does patients a disservice.” Older people may just feel the reality of them approaching death and may want to talk about it, but it may be the professionals who are afraid to bring it up.

The timeframe of a few months was agreed too, but also criticized as any timeframe may neglect different pace of person and family; it should be “on a very case-by-case basis.” A few weeks could be appropriate too. It would also depend on the stage of dementia or if already close to the end of life. It should just not be at diagnosis, and “Even if the person requests ACP at diagnosis, some time should elapse between diagnosis and ACP discussions” or end of life should only be covered “if the patient raises the issue themselves.” There was also fundamental critique to regard talking about the end of life as a separate issue because they believed the person or family will likely raise it, or that it should be integrated with discussing what people still want to do.

**Round 3**

**Section Selected recommendations regarding**ACP**in dementia: Timing - Updating**

The panel agreed upon updating ACP conversations and documents at least yearly and more frequently as the clinical condition or personal situation changes (median 5, consensus).

We received comments on feasibility in practice, and updating not always being necessary but revisiting or reviewing was recommended. We received suggestions on when to review plans, and we ask you to indicate whether you feel these should indeed trigger an update

**ACP conversations and documents should be revisited to ensure they still reflect preferences and values fully in the case of the following triggers (please check any you agree with):**

- **the person or family asking for palliative care**
- **the person or family express information needs about prognosis or future care**
- **care transition**
- **increased decline or increased fluctuation of health**
- **change in observed wellbeing or behaviour**
- **rapidly declining capacity**
- **undesirable emergency situations despite ACP**
- **high family caregiver burden or family distress**
- **family’s personal situation changes**
- **Any other trigger 1**
- **Any other trigger 2**
- **Any other trigger 3**

**Finding:**

**Triggers for updating that did not achieve a consensus, introduced in round 3 (n=84)**

| Triggers presented to the panel that did not reach the threshold for 80% endorsed | Agreement |
| --- | --- |
| *Triggers introduced in round 3 (n=84)* |  |
| change in observed wellbeing or behaviour | 69.0% |
| family’s personal situation changes | 67.9% |
| high family caregiver burden or family distress | 61.9% |


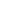


**Round 4**

**Selected recommendations regarding**ACP**in dementia: Timing**

The panel agreed upon recommendations regarding the timing of initiation of ACP, while ambiguities around providing general guidance on whether and when to refer to end of life remained. Regarding updating of ACP, several triggers were agreed upon:

These triggers achieved a consensus in the previous round

• the person or family asking for palliative care

• the person or family express information needs about prognosis or future care

• care transition

• increased decline or increased fluctuation of health

• rapidly declining capacity

• undesirable emergency situations despite ACP

A number of additional triggers for revisiting ACP plans were suggested which we would like to present to you.

**ACP conversations and documents should be revisited to ensure they still reflect preferences and values fully in the case of the following triggers**(please check any you agree with)**:**

- **rapidly declining ability of the person to communicate verbally**
- **concerns of the person or family about the process of**ACP
- **disagreement among family on goals of care or preferred treatment**
- **perception that the**ACP **plans do not adequately reflect the person’s preferences or values anymore**
- **person or family refer to the end of life**
- **person expresses considering not wanting to live anymore**
- **new services or treatment options become available and preferences may be discussed**
- **healthcare professionals’ perception of increased family distress**
- **family reporting increased caregiver burden**
- **family is no longer able to provide care or dies**

Finding:

**Triggers for updating that did not achieve a consensus, introduced in round 4 (n=89)**

| Triggers presented to the panel that did not reach the threshold for 80% endorsed | Agreement |
| --- | --- |
| family is no longer able to provide care or dies | 73.0% |
| person or family refer to the end of life | 67.4% |
| person expresses considering not wanting to live anymore | 67.4% |
| healthcare professionals’ perception of increased family distress | 58.4% |
| family reporting increased caregiver burden | 53.9% |

**Please use this space for any (brief) comments or suggestions**

Finding: 25 responses. Asking about ACP was not included as a trigger but would be the most obvious one. Some stated it would be hard to disagree with any of the presented triggers, but resource limitations imply a need to select triggers. Other panelists questioned the need to update upon triggers or found that with a natural cause of the disease, there may not be triggers to update plans.

Some criticized limiting to verbal expression as there are other ways of expressing intentions. Referring to the end of life would not automatically lead to revisiting ACP or was deemed too vague to serve as a trigger. Expressions of not wanting to live anymore or current psychological or physical pain would trigger discussions about the present and would be discussed before ACP. Likewise, family burden should trigger discussions about the (current) place of care. Interests and needs of family may complicate matters, and there were concerns that change of the family situation overrides patient’s wishes, and also questions about whose perspective is considered (person, family or staff) in the accepted trigger of plans not reflecting wishes anymore.

5. Advice from persons with young-onset dementia

(note that the numbering is different than reported in the paper where items are in order of eye-opening within categories 1 and 2)

Original question in interview guide in Dutch: "Heeft u tips voor zorgverleners over hoe zorg in de toekomst het beste te bespreken?” [Literally in English: Do you have tips for healthcare professionals how to discuss future care?]

References

Study in Flanders

Van Rickstal R, Vleminck A, Engelborghs S, Versijpt J, Van den Block L. A qualitative study with people with young-onset dementia and their family caregivers on advance care planning: A holistic, flexible, and relational approach is recommended. *Palliat Med* 2022; **36**: 964−75.

Study in the Netherlands

Maters J, van der Steen JT, Perry M, de Vugt ME, Bakker C, Koopmans RTCM. Perspectives of people with young-onset dementia on future quality of life: A qualitative interview study with implications for advance care planning. Presented at the International Psychogeriatric Association (IPA) Congress, Lisbon, Portugal, 29 June – 2 July 2023.

Survey contents

(1) Below you will find 3 recommendations based on interviews with community-dwelling persons with young-onset dementia and their family caregivers [in Flanders](https://doi.org/10.1177/02692163221090385) [reference 16 in the article]:

| a. When considering or discussing benefits of ACP, consider benefits for the person with dementia such as being involved in future care decisions, as well as for the family (for example, ACP can serve as an act of care towards the family which supports them in coping emotionally)  b. Consider discussing not only the medical aspects of ACP (e.g. medical decision-making) but also other important aspects that bring meaning as they are particularly relevant in dementia care (e.g. social care planning).  c. Use a flexible approach when discussing ACP with people with dementia and their family, as needs, capacities and perspectives of people can change throughout the dementia trajectory. |
| --- |

(2) Further, we interviewed community-dwelling persons with young-onset dementia in the Netherlands as part of the [Care4Youngdem study](https://www.trialregister.nl/trial/5834) [reference 19 in the article], also asking directly: “What would you recommend healthcare professionals on how to best discuss future care?” We summarized all recommendations below, and in most cases, we could also incorporate their feedback on it.

Adapt the setting for equal input

**d. Limit the number of participants in the conversation. Provide the right conditions for everyone to have equal input.** Conversations with several people or few breaks are difficult to follow for a person with dementia (107).

**e. Ensure the environment is optimally conducive for the conversation with that person.** This could mean that you talk while enjoying a walk outside (108-1).

Beyond medical and practical issues: who to involve

**f. Provide opportunities to discuss existential questions; refer to others if necessary.** I feel the need to talk to someone about the big questions of life, to give meaning to life. I appreciate professionals who coordinate practical matters, but I don't know whether I would be able talk to them about the big life questions (105).

**g. Don't involve too many care providers without coordinating.** "I have to be careful not to accept too much care from different sides. Everyone has their own knowledge, but they can also contradict each other. Last week I had a meeting with the psychologist, the GP and the case manager, but there is no mutual consultation. In terms of dossier management, everyone should have access to everything. Because everyone may agree with each other, but without coordination there will be no clear-cut advice” (109-1).

Set aside dedicated time, do so on time and repeatedly

**h. You, as a professional, are able to anticipate important decisions that need to be taken, so please be candid**. This provides the opportunity to take timely decisions (108-2).

**i. Schedule time to discuss uncomfortable topics**. As a professional, allow enough time, for example by scheduling the appointment at the end of the day. Scheduling a family discussion about future care needs can also be done privately, within the family environment. If you take some time, you can then feel relieved and get on with daily life, so you don't have to talk about it all the time. Indeed, "you reduce the burden on yourself by not constantly talking about it" (109-2).

**j. Structure and prioritize discussion points so that the most important issues are dealt with first; the rest comes later.** Not everything is equally relevant. You can consciously choose not to discuss certain matters now. Indeed, “we did note down all the topics, [but] also for the future. There are some topics that we don't want to discuss until Q4" (109-3).

**k. Listen carefully to really understand what someone needs.** Do not make assumptions about what someone needs, but listen carefully to the person, even if it sometimes takes more time. If needs are not immediately clear, continue to probe. Indeed, "it is important to be able to discuss everything in order to help people. Don't discuss things when you’re in a hurry, but take your time. And give people time and space to let things sink in. People can't make up their minds on the spot" (110).

The person first, next the dementia

**l. When you focus on the dementia, do not forget to enquire after the basics**. For example, people can have other than only typical dementia-related complaints or problems; they may also be living with pain that goes unnoticed when not asked about (103).

**m. Look closely at the person behind the dementia. Don't judge based on the diagnosis alone.** My social role and independence are important to me, and now that these are taken away just because the dementia diagnosis was disclosed, “we are going to discuss euthanasia with our GP; not every GP is open to this” (106).

**n. Focus on what the person wants.** Consider that some persons are more open to having conversations than others (108-3).

**Which of these recommendations do you find most valuable? (To view and choose, please navigate to previous page as needed)**

**Which of these recommendations surprise you as eye-opening, if any?**

**You may wish to reflect upon these recommendations.**

Finding: there were 26 responses. The majority of these panellists considered the recommendations as valuable and sensible. Nevertheless, some mentioned that they were not really eye-opening as they did not come up as surprising to them.

Engagement was highly valued, and panellists highlighted the importance of ACP to be implemented with a “listening attitude” and “time”, embracing a true “person-centred” approach. In what refers to the involvement of the person with dementia, capacity and family involvement were also considered, while not involving too many people in the process. Other suggestions included the inclusion of social and spiritual aspects as part of the ACP process and caution not to use ACP as just another form. Further, to anticipate and to discuss the issues before a problem arises, ACP be led by a specialist. Generalizability of the tips of persons with dementia was considered in different ways: “I feel like the notion of not involving too many people depends very much on the values and cultural context of the person. So while it surprised me and it is an important insight I'd wonder how broadly applicable it is” and “I feel that some of these recommendations (e [h in survey in Supplement] and i [n in survey in Supplement] for instance) apply to everyone not only persons with dementia.”
