# Supplementary material for: Optimizing Advance Care Planning in Dementia: Recommendations From a 33-Country Delphi Study
Source: J Pain Symptom Manage. Author manuscript; Available in PMC 2025 Oct 24. (PMC12551429; doi:10.1016/j.jpainsymman.2025.02.471)
Supplement: Supplemental A methods [file NIHMS2093260-supplement-Supplemental_A_methods.docx]

**Supplement A. Methods**

**Achieving a consensus on ACP in dementia****:**

**Study design and panel**

Supplemental material (1 of 2) with the article “Optimizing advance care planning in dementia: Recommendations from a 33-country Delphi Study”

1. Delphi study phases

2. Anticipated and actually conducted rounds, timelines and contents of the online Delphi rounds

3. Building up the evidence and consensus based clinical guidance on ACP in dementia: Methods and sources

4. Recruitment of the panel

5. Flow chart participation Delphi expert panel and response per survey round

6. Task force and panelists’ characteristics

Numbers 1, 2, 5 and 6 reproduced from: van der Steen JT, Nakanishi M, Van den Block L, Di Giulio P, Gonella S, In der Schmitten J, Sudore RL, Harrison Dening K, Parker D, Mimica N, Holmerova I, Larkin P, Martins Pereira S, Rietjens JAC, Korfage IJ; European Association for Palliative Care (EAPC). Consensus definition of advance care planning in dementia: A 33-country Delphi study. *Alzheimers Dement*. 2024;20:1309-1320. Open access at <https://doi.org/10.1002/alz.13526>

Number 3 represents a more detailed version of Fig. 2 in the article “Optimizing advance care planning in dementia: Recommendations from a 33-country Delphi Study”

Number 4 reproduced from:

Nakanishi M, Martins Pereira S, Van den Block L, Parker D, Harrison-Dening K, Di Giulio P, In der Schmitten J, Larkin PJ, Mimica N, Sudore RL, Holmerová I, Korfage IJ, van der Steen JT; European Association for Palliative Care. Future policy and research for advance care planning in dementia: consensus recommendations from an international Delphi panel of the European Association for Palliative Care. *Lancet Healthy Longev*. 2024;5:e370-e378.

Open access at <https://doi.org/10.1016/S2666-7568(24)00043-6>

The full pre-registered protocol is directly accessible at: <https://osf.io/u7pkx>

van der Steen JT, Rietjens J, Korfage I. Research protocol for ‘European Association for Palliative Care (EAPC) Delphi study on advance care planning in dementia: Conceptualisation and recommendations for practice, policy and research. Leiden: LUMC, June 2021. <https://osf.io/tjyra> See heading ‘Study design.’” Registered with Open Science Framework (OSF) at 7 September 2021. Disembargoed at 2 September 2023. Registered 7 September 2021 with the WHO international clinical trials registry platform Netherlands Trial Register number NL9720.

**1. Table: Delphi study phases**.

| Phase – round (time) | Activity | Design; input | Contributors |
| --- | --- | --- | --- |
| Phase A – Preparation  (February 2019 – September 2021) | Prepare content (up to November 2020) and protocol identify candidate panelists (up to September 2021) Delphi study | Content development: qualitative synthesis of general ACP frameworks - previous generic EAPC ACP conceptualization of 41 elements (Rietjens et al., 2017), literature review, and task force member expertise.  Study protocol development: previous EAPC Delphi studies (Rietjens et al., 2017; van der Steen et al., 2014) and reporting guideline (Jünger et al., 2017) | Task force members (15^a^ members from 12 countries; the members involving 3 additional experts in their countries). Selected analytic tasks were conducted by a core team of 5 members only. |
| Phase B – Consensus round 1  (30 September 2021 – 22 October 2021) | Online survey | Quantitative agreement scale and qualitative open-ended items | Panel of 107 experts from 33 countries (97 participating in this round) |
| in-between rounds 1 and 2 | Analyses of survey results round 1; setting up content for round 2 | Feedback from the panel in round 1 | Task force members (14^a^ members from 12 countries; 1 additional expert) |
| Phase B – Consensus round 2  (15-17 December 2021 – 17 January 2022) | Online survey | Quantitative agreement scale and qualitative open-ended items | Panel of 107 experts from 33 countries (86 participating in this round) |
| analyses in-between rounds 2 and 3 | Analyses of survey results round 2; setting up content for round 3 | Feedback from the panel in round 2 | Task force members (14 members from 12 countries; 1 additional expert) |
| Phase B – Consensus round 3 (7-8 March 2022 – 1 April 2022) | Online survey | Quantitative agreement scale and qualitative open-ended items | Panel of 107 experts from 33 countries (87 participating in this round) |
| analysis in-between rounds 3 and 4 | Analyses of survey results round 3; setting up content for round 4 | Feedback from the panel in round 3 | Task force members (14 members from 12 countries; 1 additional expert) |
| Phase B – Consensus round 4 (19-20 May 2022 – 29 June 2022) | Online survey | Quantitative agreement scale and qualitative open-ended items | Panel of 107 experts from 33 countries (87 participating in this round) |
| analysis after round 4 (summer 2022) | Analyses of survey results 4 – decision to conclude survey | Feedback from the panel in round 4 | Task force members (14 members from 12 countries; 1 additional expert) |
| Phase C – Review and approval (February 2023) | EAPC Board review | Article white paper | EAPC Board |

^a^We lost our appreciated task force member Dr Kathryn Higher who died during Phase A.

**References**

Jünger S, Payne SA, Brine J, Radbruch L, Brearley SG. Guidance on Conducting and REporting DElphi Studies (CREDES) in palliative care: Recommendations based on a methodological systematic review. Palliat Med. 2017 Sep;31(8):684-706. doi: 10.1177/0269216317690685.

Rietjens JAC, Sudore RL, Connolly M, van Delden JJ, Drickamer MA, Droger M, van der Heide A, Heyland DK, Houttekier D, Janssen DJA, Orsi L, Payne S, Seymour J, Jox RJ, Korfage IJ; European Association for Palliative Care. Definition and recommendations for advance care planning: an international consensus supported by the European Association for Palliative Care. Lancet Oncol. 2017 Sep;18(9):e543-e551. doi: 10.1016/S1470-2045(17)30582-X.

van der Steen JT, Radbruch L, Hertogh CM, de Boer ME, Hughes JC, Larkin P, Francke AL, Jünger S, Gove D, Firth P, Koopmans RT, Volicer L; European Association for Palliative Care (EAPC). White paper defining optimal palliative care in older people with dementia: a Delphi study and recommendations from the European Association for Palliative Care. Palliat Med. 2014 Mar;28(3):197-209. doi: 10.1177/0269216313493685.

**2. Table: Anticipated and actually conducted rounds, timelines and contents of the online Delphi rounds.**

*(italics: feedback to the panel with improved contents or on reaching a consensus;* highlighted: aim of conceptualization of advance care planning in dementia addressed in ****** this article*)*

| Round | Anticipated rounds – Protocol text (OSF, 2 September 2021) | Actually conducted rounds 30 September 2021 – 29 June 2022 | Explanation of deviations |
| --- | --- | --- | --- |
| 1 | First round – planned September 2021  • a definition of advance care planning in dementia adapted from the EAPC definition of advance care planning for persons with capacity;  • three domains that should capture the main specifics for ACP in case of dementia and a model visualizing how the domains may be connected;  • a brief selection of recommendations;  • a palliative goals of care model (new Figure detailing on psychosocial care goals);  • a few items about the respondents. | First round 30 September 2021 – 22 October 2021  • contents as planned (see left column). The brief selection of recommendations concerned recommended elements of ACP and timing  • comparison of palliative goals of care models (compared with dementia white paper Figure; had been planned for round 2). | Apart from already including content planned for round 2, there were no deviations to the planned contents of the first round. |
| 2 | Second round projected November 2021  • abbreviated and lay definitions;  • any revised elements with anonymized group feedback;  • comparison of palliative goals of care models (compared with dementia white paper Figure);  • all recommendations except those presented in the first round that achieved a consensus immediately | Second round 15-17^a^ December 2021 – 17 January 2022  • abbreviated definition;  *• revised model visualizing how the domains may be connected with feedback;*  • *a palliative goals of care model with feedback;*  • *selection of recommendations: recommended elements of ACP* and any difference with young-onset dementia;  • *selection of recommendations: timing of ACP.* | (Subsequent rounds were based on interim analyses and the panelists’ feedback in the previous round.)  No lay definition was developed and presented as we considered the expert panel unfit to evaluate it.  In addition to the abbreviated definition, we decided to present elements of the full definition that panelist may prefer to retain in the abbreviated definition.  Any difference with young-onset dementia planned in round 3 was moved to round 2 for the opportunity to link to an overview of elements of ACP. |
| 3 | Third round projected January 2022  • the full set of recommendations, visualizing which have been accepted already, revised or newly added if any;  • overall importance and best practice statements;  • any difference with young-onset dementia;  • possible gaps in international policy and research. | Third round 7-8^a^ March 2022 – 1 April 2022  *• three domains that should capture the main specifics for ACP in case of dementia and a model visualizing how the domains may be connected;*  • *selection of recommendations: recommended elements of ACP;*  • *selection of recommendations: timing;*  • selection of recommendations: roles and tasks;  • selection of recommendations: recommended elements of policy and regulation. | Possible gaps in international policy and research was moved to the next round to reduce the length of the third-round survey. |
| 4 | Fourth or also fifth round (before summer 2022)  • views from persons with dementia phrased as tips for professionals, if available on time (for comments only, not for revision);  • list of summarized goals, benefits and limitations of ACP in dementia;  • any items that have not reached a consensus while further revision is expect to affect ratings;  • any new items based on the panelists feedback. | Fourth round 19-20^a^  May 2022 – 29 June 2022  • *we briefly informed the panel on reaching a consensus on the definition and the visualization of the three issues*  • *selection of recommendations: timing;*  • *selection of recommendations: recommended elements of policy and regulation*;  • possible gaps in international policy and research;  • views from persons with dementia phrased as tips for professionals ;  • list of summarized goals, benefits and limitations of ACP in dementia;  • another item about the respondent: experience in ACP in groups other than in persons with dementia;  • evaluation of personal experience participating in the 4-round survey. | We decided to a more extensive fourth and last round.  The list of summarized goals, benefits and limitations of ACP in dementia referred to recommended evaluation of ACP and covered a quite extensive list. We expanded list of possible elements from Rietjens et al. 2017 with possible goals, benefits and limitations from the literature. We also asked about and generalizability to persons without or with limited capacity more generally. To limit the length of the survey, we did not inform the panel on reaching a consensus on roles and tasks and recommended elements*.* |

^a^Invitations to later rounds included personalized feedback and finishing this task for all participating panelists took the researcher and research assistant more day.


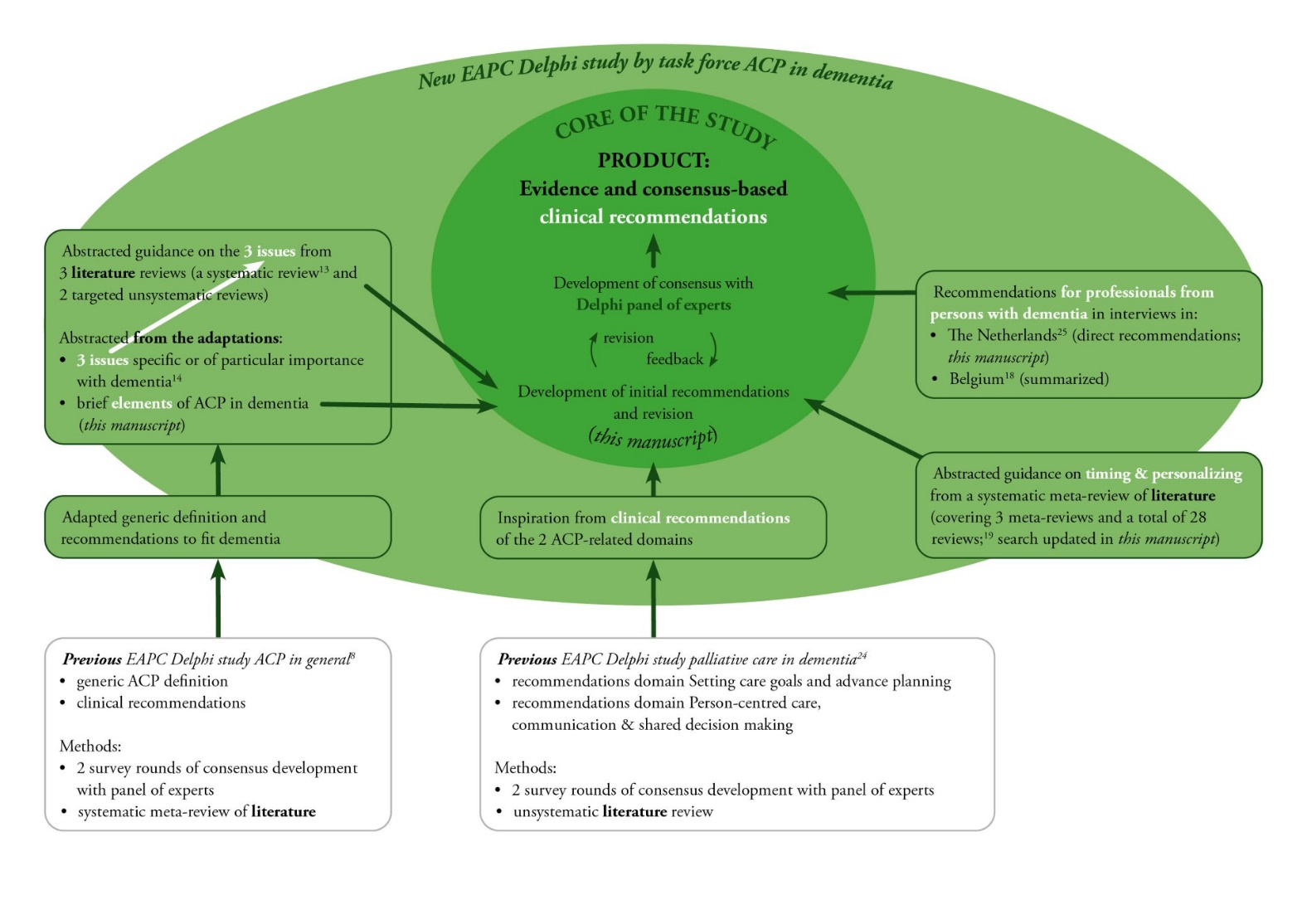
**3. Fig.:** **Building up the evidence and consensus based clinical guidance on ACP in dementia: Methods and sources** (detailed version of Fig.2 in the article).

The evidence and consensus-based clinical recommendations represent the new end product presented in this manuscript, adding to other products of the task force answering the other research questions: a definitional framework^14^ and recommendations for policy & research^20^

The 4 literature sources (in bold) line up with Box 1 Integration of evidence in the consensus-building process.

ACP= advance care planning

14. van der Steen JT et al.; EAPC. Consensus definition of advance care planning in dementia: A 33-country Delphi study. Alzheimers Dement. 2024;20:1309-1320.

20. Nakanishi M et al.; EAPC. Future policy and research for advance care planning in dementia: consensus recommendations from an international Delphi panel of the European Association for Palliative Care. Lancet Healthy Longev. 2024;5:e370-e378.

**4. Fig.: Integration of evidence in the consensus-building process**

| **Literature** was included building up an accumulating evidence base through multiple layers of meta syntheses over 2020 and 2021 which was integrated with **expert views** elicited in the 2021 and 2022 survey rounds.   - **We built upon earlier Delphi studies based on literature and transparent expert view** - The European Association for Palliative Care (EAPC) task force of experts in advance care planning (ACP) in dementia started with adapting generic recommendations for ACP (Rietjens et al.)^8^ to fit with dementia. The generic recommendations had been developed in an earlier Delphi study of the EAPC that was based on a systematic literature search in 2016 which identified 89 reviews and a meta-synthesis, position papers and other literature on ACP. - Additional recommendations were inspired by literature assembled by another earlier related EAPC Delphi study on palliative care in dementia (van der Steen et al.).^24^ - **We used newer literature specifically on ACP in dementia** - In parallel, for three issues identified as specific for dementia from adapting the generic guidance^8^ late 2020, to create key recommendations in consultation with experts in a structured manner, three taskforce subgroups reviewed the literature. Two rapid targeted searches were conducted (capacity and family, early 2021^14^) and a systematic scoping review on engagement and communication with persons with dementia (started late 2020)^13^ for which the recommendations and underpinnings seemed sparser compared to the issues of capacity and family. - A total of 24 literature reviews covered by the three available meta-reviews early 2021^19^ inspired the task force in the development of initial recommendations. No new meta-reviews were found with a comprehensive database search up to 30 November 2023. We then searched the PubMed, Web of Science, PsycINFO, Scopus and CINAHL databases with keywords ((dementia) AND (advance care planning)) AND ((meta-review) OR (umbrella review) OR (meta-analysis) OR (meta-synthesis) OR (overview) OR (review of reviews) OR (review of systematic reviews)) which resulted in 381 unique records of any reviews and 8 review protocols screened by two researchers. Of the three meta-reviews identified, two had been identified by the original and updated searches while one meta-review by collaborators was still in progress, and the authors of an umbrella review protocol reported no progress.   **Relevance of the new Delphi study based on findings in the literature**   - ***Key challenges in clinical practice*** ***were reported in the reviews.*** These revolved around diminishing decision-making capacity over time and moral dilemmas and challenges in implementation. For example, advance directives may not be a panacea in cases of dementia as they cannot always be updated with changing situations. Further, a number of reviews identified numerous barriers on part of the person with dementia and family, and healthcare professionals to initiate and communicate on ACP and to broach the subject of end of life. - ***How to optimally conduct ACP* *in dementia was underpinned by limited evidence***. Reviews on ACP in dementia were being published at an increasing and greater pace than results of trials. This indicated the relevance in synthesizing fragmented knowledge on how to conduct conversations about future care, preferably at a time the person can still communicate preferences. |
| --- |

**5. Table: Recruitment of the panel:** **Source of identification and characteristics of potential candidates (n=169)**

|  | **Group** | **n** |
| --- | --- | --- |
| Source of identification (more possible) | Networks of EAPC ACP in dementia taskforce (connections and via-via suggestions from national and international organisations, conferences and research groups for dementia and palliative care) | 101 |
|  | Participants in previous Delphi study | 42 |
|  | Palliative care in dementia | 24 |
|  | Generic ACP | 18 |
|  | Dementia-related organisations’ websites | 18 |
|  | PubMed search using keywords of ‘dementia’ and ‘advance care planning’ with targeting authors from Africa, Asia other than Japan, and the South and Central Americas | 24 |
| Profession (more possible) | Physician, physician assistant or nurse practitioner | 79 |
|  | Nurse (any level) | 36 |
|  | Psychologist | 22 |
|  | Ethicist | 10 |
|  | Policy/administration | 27 |
|  | Social worker | 8 |
|  | Epidemiologist (self-report by respondents) | 4 |
|  | Spiritual counsellor | 3 |
|  | Other, including epidemiologist, sociologist, occupational therapist, lawyer, economist, gerontologist, philosopher, speech-language therapist, physiotherapist and pharmacist | 18 |
| Country classification by continent | Western country: Europe, Northern Americas, Australasia and alternating between continents | 129 |
|  | Non-western country: Asia, Middle East, Africa, Southern and Middle Americas | 40 |
| Country classification by income | HIC | 141 |
|  | LMIC | 28 |

Legend. ACP, advance care planning. EAPC, European Association for Palliative Care. HIC, high income country. LMIC, low- and middle-income country. A total of 169 candidates from 43 countries; of 178 candidates from 46 countries invited, 9 were excluded as invitation email not deliverable (n=8) or did not receive invitation in time (n=1). Multiple sources of identification and multiple professions could apply for one candidate: the sum of numbers is not equal to the total number of candidates. Profession of respondents (n=107) is based on self-report in the survey, while that of non-respondents (n=62) was based on publicly available information used to aim at recruiting a diverse sample. The response rate was significantly lower among candidates from non-western countries (4.5%) compared with those from western countries (68.2%; χ^2^(1)=5.64, P=.018). Similarly, the response rate of candidates from LMICs (42.9%) was significantly lower than those from HICs (67.4%; χ^2^(1)=6.05, P=·.014). Caution: given that candidates from non-western countries and LMICs were invited more often after identifying them through internet searches rather than through personal connections, which is generally a less effective recruitment strategy, we cannot interpret the significantly different response rates in terms of response bias.

**6.** **Table: Task force and panelists’ characteristics.**

|  | **Task force (n=14)**^a^ | **Panel (n=107)** | |
| --- | --- | --- | --- |
|  | **n or mean (SD)** | **n** | **% or mean (SD)** |
| **Continent of residence (panelists: 33 countries), %**  Europe (EuroVoc;^b^ 18 countries; panelists only: 17) |  |  |  |
| Western Europe (7) | 7 | 35 | 32.7 |
| Southern Europe (3) | 2 | 15 | 14.0 |
| Northern Europe (4) | 0 | 7 | 6.5 |
| Eastern and Central Europe (4; panelists only: 3) | 2 | 6 | 5.6 |
| Americas (4 countries) |  |  |  |
| Northern Americas (2) | 1 | 17 | 15.9 |
| Southern and Middle Americas (2) | 0 | 3 | 2.8 |
| Asia (6 countries) | 1 | 11 | 10.3 |
| Australasia (2 countries) | 0 | 6 | 5.6 |
| Middle East (2 countries) | 0 | 3 | 2.8 |
| Africa (2 countries) | 0 | 2 | 1.9 |
| Alternating between continents (4 countries, all with other participants) | 0 | 2 | 1.9 |
| **Gender, %** |  |  |  |
| woman | 11 | 74 | 69.2 |
| man | 3 | 33 | 30.8 |
| other | 0 | 0 | 0 |
| prefer not to say | 0 | 0 | 0 |
| **Age, mean number of years (SD)** | 55.0 (8.3) | 104 | 52.0 (12.1) |
| **Professional experience, mean number of years (SD)** | 29.9 (10.0) | 103 | 24.4 (11.8) |
| **Profession (more possible),%** |  |  |  |
| medical | 4 | 53 | 49.5 |
| physician | 4 | 52 | 48.6 |
| physician assistant or nurse practitioner | 0 | 1 | 0.9 |
| nurse (any level) | 7 | 21 | 19.6 |
| psychologist | 2 | 12 | 11.2 |
| ethicist | 1 | 9 | 8.4 |
| policy / administration | 2 | 8 | 7.5 |
| social worker | 0 | 5 | 4.7 |
| epidemiologist | 2 | 4 | 3.7 |
| spiritual counsellor | 0 | 3 | 2.8 |
| other, e.g. sociologist, occupational therapist, lawyer, economist | 3 | 12 | 11.2 |
| **Researcher, past and recent activity,%** |  |  |  |
| very active researcher  (leading research and publishing extensively) | 12 | 53 | 49.5 |
| researcher but not particularly active  (contributing to research and some publications) | 2 | 35 | 32.7 |
| PhD student or early career researcher | 0 | 13 | 12.1 |
| not a researcher | 0 | 6 | 5.6 |
| **Specific expertise in ACP in dementia %** |  |  |  |
| yes, ACP in dementia specifically  no, ACP, or dementia, or both but not combined | 11  3 | 60  44 | 57.7  42.3 |
| **Expertise in ACP in other populations or the general population, %**^c^  yes  no such experience | 11  3 | 66  21 | 75.9  24.1 |
| **Personally experienced a family member or friend, %**  having advanced dementia at the end of their life, yes  no such experience | 12  2 | 72  30 | 70.6  29.4 |
|  |  |  |  |

^a^We started the task force with 15 members but lost one member who died in phase 1. Data on n=14 are complete. Task force roles included chair (n=2) who were also core team and subgroup member, other core team and subgroup member (n=2), subgroup member (n=7), subgroup member and EAPC link person (n=1) or member with no special task (n=2).

Roles: two chairs who are also core team and subgroup member, 2 core group and subgroup member, 1 core group member only, 4 subgroup members, 4 regular members, and 1 EAPC link person.

^b^EuroVoc multilingual thesaurus maintained by the Publications Office of the European Union.

^c^n=87; refers to experience through practice, research or policy asked in fourth round whereas all other characteristics were asked in first round which allowed for following up on any missing responses.
